# Systematic review on chronic non-communicable disease in disaster settings

**DOI:** 10.1186/s12889-022-13399-z

**Published:** 2022-06-21

**Authors:** Christine Ngaruiya, Robyn Bernstein, Rebecca Leff, Lydia Wallace, Pooja Agrawal, Anand Selvam, Denise Hersey, Alison Hayward

**Affiliations:** 1 Department of Emergency Medicine, Yale University, 464 Congress Avenue, Suite #260, New Haven, CT 06519 USA; 2 Department of Chronic Disease Epidemiology, Yale School of Public Health, New Haven, CT USA; 3 Department of Emergency Medicine, Mayo Clinic, Rochester, Minnesota USA; 4 Director, Dana Medical Library, University of Vermont, Burlington, VT USA; 5 Division of Global Emergency Medicine, Department of Emergency Medicine, The Warren Alpert Medical School of Brown University, Providence, USA

**Keywords:** NCDs, Non communicable diseases, Disaster, Warfare and armed conflicts, Cardiovascular disease, Diabetes mellitus, Chronic obstructive pulmonary disease, Asthma, Disaster medicine, Cancer

## Abstract

**Background:**

Non-communicable diseases (NCDs) constitute the leading cause of mortality globally. Low and middle-income countries (LMICs) not only experience the largest burden of humanitarian emergencies but are also disproportionately affected by NCDs, yet primary focus on the topic is lagging. We conducted a systematic review on the effect of humanitarian disasters on NCDs in LMICs assessing epidemiology, interventions, and treatment.

**Methods:**

A systematic search in MEDLINE, MEDLINE (PubMed, for in-process and non-indexed citations), Social Science Citation Index, and Global Health (EBSCO) for indexed articles published before December 11, 2017 was conducted, and publications reporting on NCDs and humanitarian emergencies in LMICs were included. We extracted and synthesized results using a thematic analysis approach and present the results by disease type. The study is registered at PROSPERO (CRD42018088769).

**Results:**

Of the 85 included publications, most reported on observational research studies and almost half (48.9%) reported on studies in the Eastern Mediterranean Region (EMRO), with scant studies reporting on the African and Americas regions. NCDs represented a significant burden for populations affected by humanitarian crises in our findings, despite a dearth of data from particular regions and disease categories. The majority of studies included in our review presented epidemiologic evidence for the burden of disease, while few studies addressed clinical management or intervention delivery. Commonly cited barriers to healthcare access in all phases of disaster and major disease diagnoses studied included: low levels of education, financial difficulties, displacement, illiteracy, lack of access to medications, affordability of treatment and monitoring devices, and centralized healthcare infrastructure for NCDs. Screening and prevention for NCDs in disaster-prone settings was supported. Refugee status was independently identified both as a risk factor for diagnosis with an NCD and conferring worse morbidity.

**Conclusions:**

An increased focus on the effects of, and mitigating factors for, NCDs occurring in disaster-afflicted LMICs is needed. While the majority of studies included in our review presented epidemiologic evidence for the burden of disease, research is needed to address contributing factors, interventions, and means of managing disease during humanitarian emergencies in LMICs.

**Supplementary Information:**

The online version contains supplementary material available at 10.1186/s12889-022-13399-z.

## Background

Non-communicable diseases (NCDs) constitute the leading cause of mortality globally, accounting for 70% of deaths worldwide [1]. This percentage is projected to rise in the next fifteen years, with the steepest increase in morbidity and mortality from NCDs projected to occur in Low and Middle-Income Countries (LMICs). The World Health Organization (WHO) projects a 10% rise in mortality in Africa from NCDs in from 2015 to 2030 [2]. This rise in NCDs in LMICs coincides with an increasing burden of humanitarian disasters [3].

The International Red Cross defines a disaster as: “a sudden, calamitous event that seriously disrupts the functioning of a community or society and causes human, material, and economic or environmental losses that exceed the community’s or society’s ability to cope using its own resources” [4], and can be divided into: mitigation, preparedness, response, and recovery phases [5]. The United Nations Office for Disaster Risk Reduction (UNISDR) recorded over 1.35 million people killed by natural hazards between 1997–2017, with disproportionate mortality in LMICs [6]. Poverty, rapid urbanization, inadequate infrastructure, and underdeveloped disaster warning and health systems are all contributors to morbidity and mortality in disasters [6, 7].

According to the UNHCR Global Trends Report, an unprecedented 79.5 million people are estimated to have been displaced from their homes as internally displaced persons (IDPs) or refugees in 2019—the largest figure ever recorded [8]. The scale of humanitarian disasters has increased in recent decades for two primary reasons. Firstly, the frequency and ferocity of natural disasters are increasing due to climate change [9]. Secondly, the number of refugees, displaced persons, and migrants are at an all-time high due to the unprecedented refugee crises in Syria, Iraq, and the Democratic Republic of Congo [10]. Disasters may directly exacerbate NCDs through effects such as increased stress levels [11], exposures such as inhalation of substances that trigger worsening of pulmonary disease [12], and exacerbation of underlying disease secondary to limited access to care [13].

Despite the growing burden of humanitarian crises with increasing populations at risk for morbidity and mortality from NCDs, primary focus on the topic is lagging. It is essential to better understand the effect of disasters on NCDs in LMICs as the mortality and morbidity are projected only to increase given climate change and population growth in vulnerable areas [14]. In this context, we conducted a systematic review on the effect of humanitarian disasters on NCDs in LMICs assessing epidemiology, interventions, and treatment. While a limited number of articles have reviewed interventions for NCD management [15, 16], a single NCD disease type [17, 18], or a single geographic region in disaster settings [18–21], to our knowledge, this is the first systematic review of its kind cross-cutting both regions and disease type. Our aims are to guide allocation of resources, future research, and policy development.

## Methods

An experienced medical librarian performed a comprehensive search of multiple databases after consultation with the lead authors and a Medical Subject Heading (MeSH) analysis of key articles provided by the research team.

### Eligibility criteria

In each database, we used an iterative process to translate and refine the searches. English, Arabic and French language articles were eligible based on these languages being spoken frequently in LMICs, our team’s language capabilities, and so as not to limit solely to English language articles and potential reporting bias as a result [22]. The formal search strategies used relevant controlled vocabulary terms and synonymous free text words and phrases to capture the concepts of noncommunicable, chronic and noninfectious diseases, and different types of humanitarian emergencies including natural disasters, armed conflicts, terrorism, and failed states (see Additional file 1).

### Information sources

The databases searched were MEDLINE (OvidSP 1946-August Week 2 2015), MEDLINE (PubMed, for in-process and non-indexed citations), Social Science Citation Index, and Global Health (EBSCO).

### Search strategy

We included studies conducted in LMICs investigating non-communicable diseases in the context of humanitarian emergencies; LMICs were categorized as outlined by The World Bank [23]. Studies conducted in high income countries (HICs) and review articles were excluded. Mental health and associated terms were not included in this review given evidence on the disease burden in existing literature [24–28] and our own research question which sought to address the leading four NCDs (cardiovascular disease, diabetes, cancer and chronic respiratory disease) as outlined by the WHO [29]. No other restrictions on study type were applied. The original searches were run August 10, 2015 and were rerun on December 11, 2017. No date restrictions were applied such that any publication prior to this date was potentially eligible for inclusion. The full strategy for PubMed is available in the Additional file 1. The study is registered at PROSPERO (CRD42018088769).

### Selection process

**Fig. 1:**
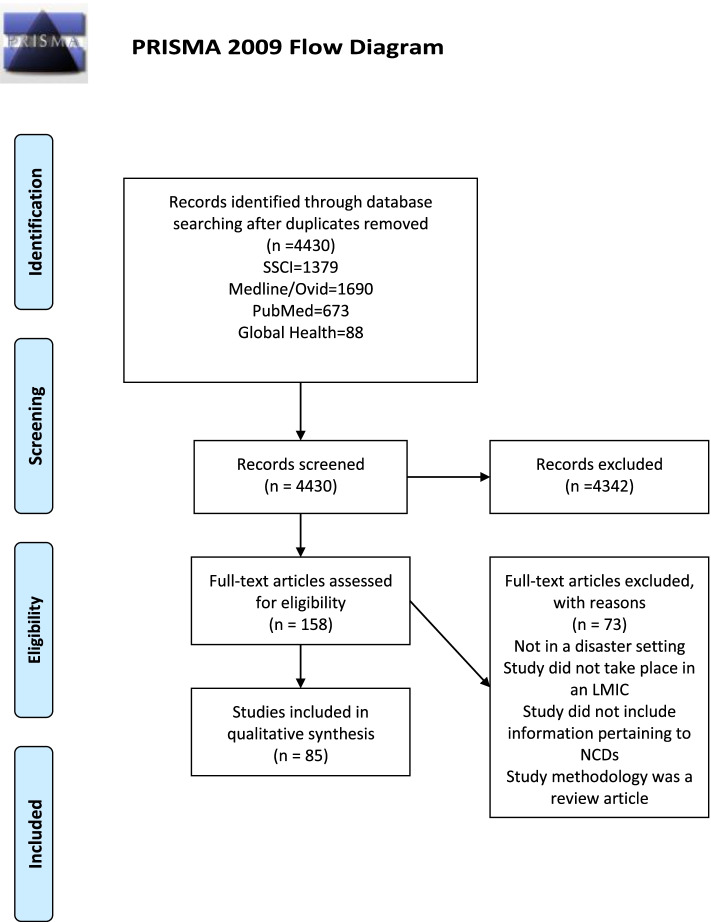
PRISMA Flow Diagram

Retrieved references were pooled in EndNote and de-duplicated to 4,430 citations. Two separate screeners independently evaluated the titles, abstracts and full text of the eligible articles (RB and LW), with vetting by a third reviewer (CN). The flowchart per PRISMA is presented in Fig. 1. An assessment of the risk of bias of included studies is provided in tabular format in the Additional file 1.

### Study risk of bias assessment

Bias was evaluated using the Newcastle–Ottawa scale for assessing risk of bias given majority observational studies in our findings [30].

## Results

**Fig. 2:**
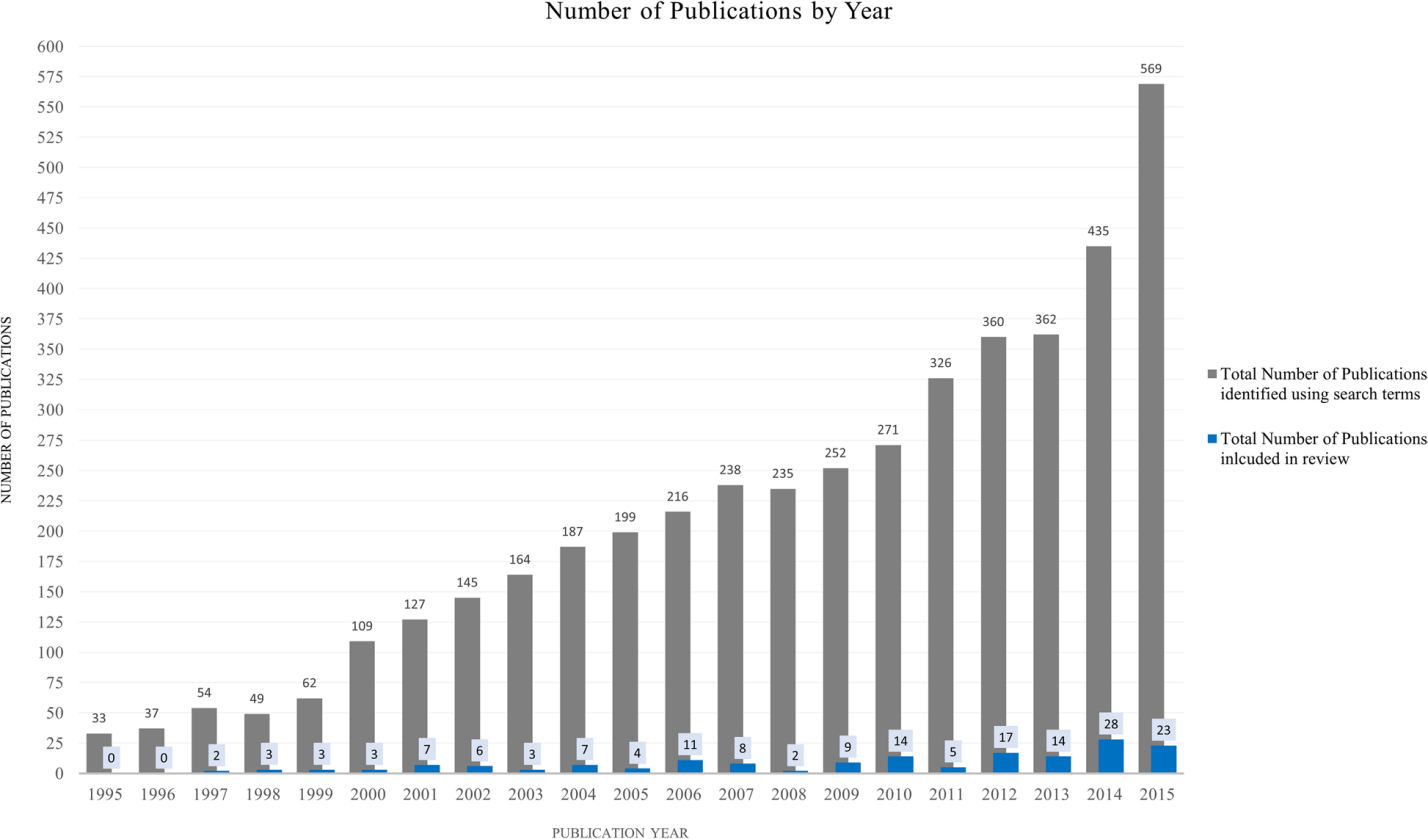
Number of Publications by Year

**Fig. 3:**
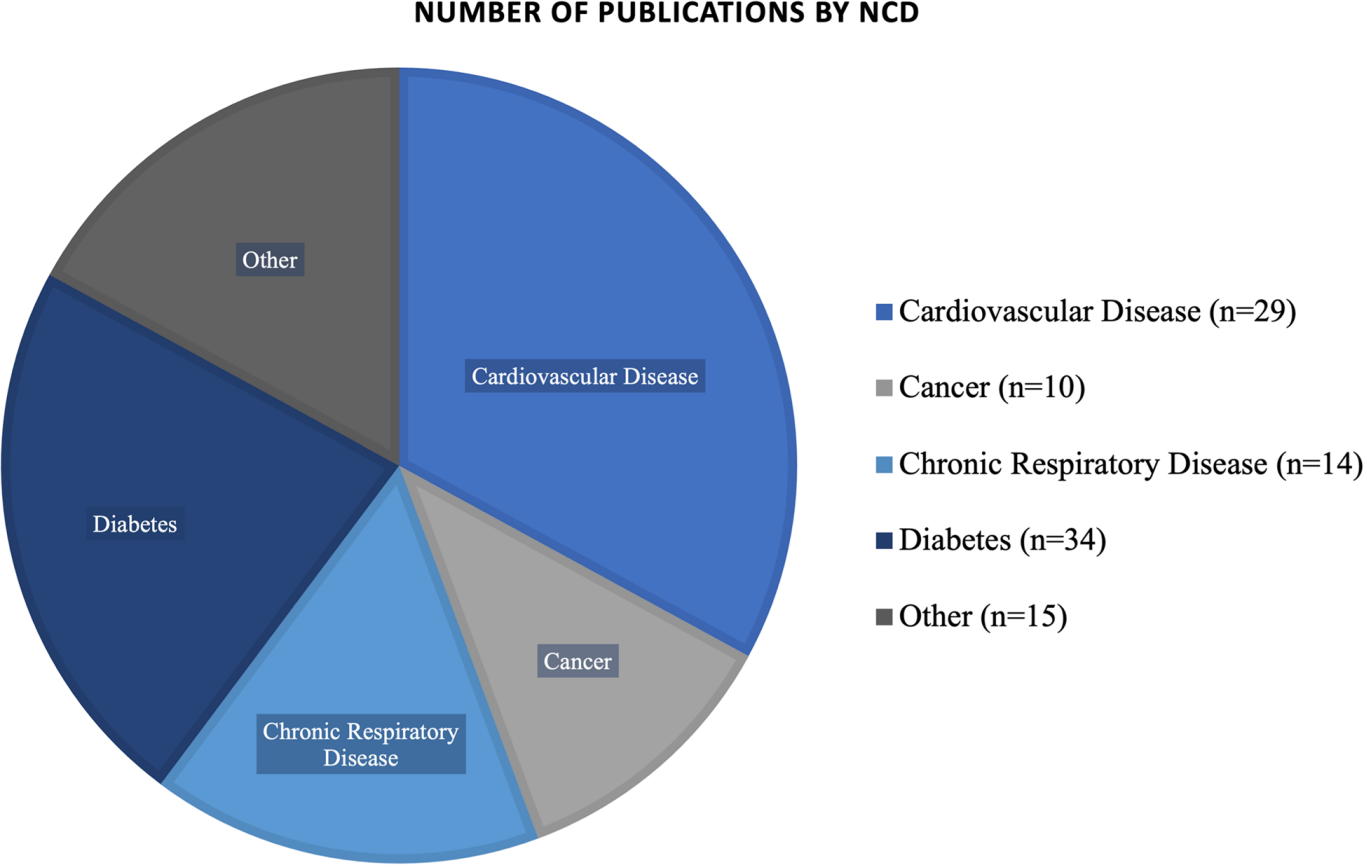
Number of Publications by NCD

**Fig. 4:**
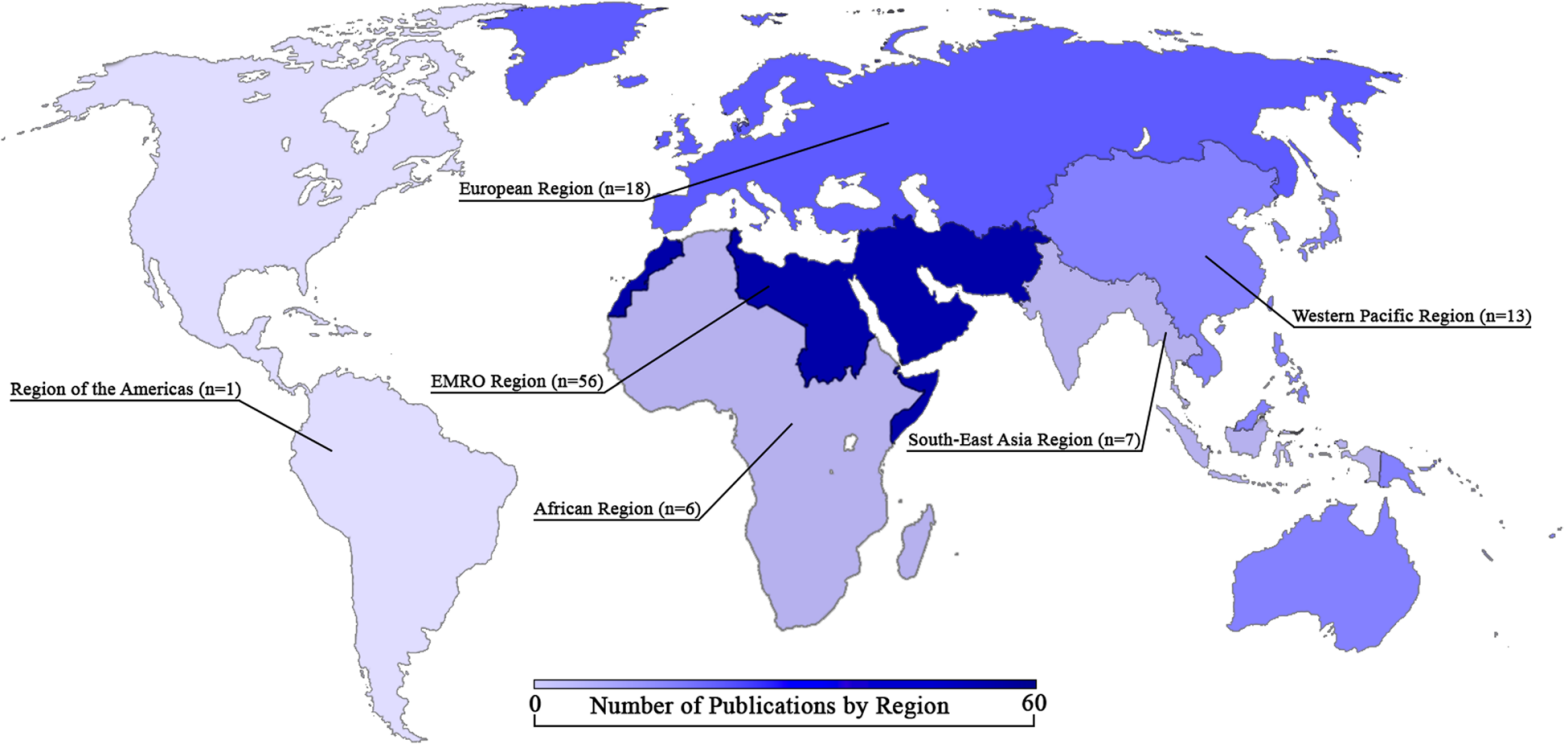
NCD Heat Map

We retrieved a total of 4,430 references. Four thousand three hundred forty-two studies were excluded by title or abstract, and 158 articles were read in full. Out of the studies screened by full text, 85 studies are included in the final thematic analysis (Tables 1, 2, 3, 4, 5, 6, 7, 8, 9 and 10; Fig. 2), with increasing publications on the topic over time (Fig. 2). For ease of review, we have presented the results by disease type (Tables 1, 2, 3, 4 and 5; Fig. 3) including summaries on study type as well as epidemiology of disease addressed. We felt that the study design would be relevant, in addition to the disease focus, in order to elucidate opportunities for future research based on study approaches that were lacking. The diseases types are split into five categories, which consist of the lead four NCDs in order of burden [29]: cardiovascular disease (CVD), cancer, chronic respiratory disease, diabetes, and a section on other NCDs (defined as those identified in our results that assessed NCDs not fitting into one of the lead four categories). We have also grouped the articles by region, and those results have been presented in tabular format and graphically (Tables 6, 7, 8, 9 and 10; Fig. 4). We present the results on interventions in detail elsewhere [31].

**Table 1:** Characteristics of included publications by disease type: Cardiovascular Disease

	**Country/** **Territory of Interest**	**WHO region**	**Type of study**	**Target Population**	**Years of observation**	**Number of study participants**	**Major findings**
Abukhdeir (2013) [32]	Palestinian Territories: West Bank/Gaza	EMRO	Cross sectional	Palestinian households in the West Bank and Gaza Strip	May 2004—July 2004	4,456 households in the West Bank and 2118 in the Gaza Strip	Being a refugee was a significant risk factor for CVD while being married/engaged or divorced/separated/ widowed was a risk factor for hypertension. Non-refugees were 46% less likely to have CVD than refugees. Gender was a risk factor for hypertension with females being 60% more likely to have hypertension than males. Age was a significant risk factor for hypertension and CVD(*p* < 0.0001)
Ahmad (2015) [33]	Syria	EMRO	Situational analysis using document analysis, key informant interviews, and direct clinic observation	Syrian national health system	October 2009 -August 2010	53 semi-structured interviews	The rebuilding of a post-conflict heath care system in Syria may benefit from insights into the structural problems of the pre-crisis system. Weaknesses that existed before the crisis are compounded by the current conflict
Armenian (1998) [34]	Armenia	Europe	Retrospective cohort	Employees of the Armenian Ministry of Health and their immediate families who survived the 1988 Earthquake in Armenia	1990–1992	35,043 persons (7,721 employees who had survived the disaster and their family members)	The nested case–control analysis of 483 cases of newly reported heart disease and 482 matched non-heart-disease controls revealed that people with increasing levels of loss of material possessions and family members had significant increases in heart disease risk (OR for “loss scores” of 1, 2, and 3 were 1.3, 1.8, and 2.6, respectively)
Ben Romdhane (2015) [35]	Tunisia	EMRO	Situational analysis	Tunisian national health system	2010	12 key informants were interviewed and eight documents were reviewed	Weaknesses that existed before the 2011 Revolution (Arab Spring) were compounded during the revolution. This study was conducted prior to political conflict but written post-conflict. Growth of the private sector fostered unequal access by socioeconomic status and reduced coordination and preparedness of the health system
Bergovec (2005) [36]	Bosnia and Herzegovina	Europe	Retrospective chart review	The population that lived in Mostar and the nine neighboring districts prior to the Bosnian War(1992–1995)	Five consecutive years (1987–1991) before the war and 5 consecutive years (1992–1996) during the war were analyzed	182,000 persons per the 1991 census	There was a wartime increase in acute myocardial infarctions(AMI) for the total population (*p* = 0.025). There was a statistically significant increase (*p* = 0.001) in the total number of unstable angina pectoris(UA) cases during the war (185 cases, compared with 125 prewar cases). Females experienced a statistically significant increase in UA and AMI(*p* = 0.001, 0.007 respectively) whereas the increase among men was not statistically significant (*p* = 0.072, *p* = 0.354 respectively)
Chen (2009) [37]	China	Western Pacific	Case series	Adults who were in the West China Hospital on the day of the 2008 Sichuan (Wenchuan) earthquake	May 2008	11 patients	Mean blood pressure and heart rate increased immediately after the earthquake, regardless of gender or pre-existing hypertension. BP gradually declined within 6 h after the earthquake and increased again during aftershocks. Circadian variation was absent in all cases
Ebling (2007) [38]	Croatia	Europe	Multipart study including both a retrospective cohort study and an uncontrolled before-after study	Refugee-returnees of the 1991–1992 war operations in Eastern Slavonia from Osjek-Baranga County, Croatia	2003	retrospective cohort study: 589 participants uncontrolled before-after study 202 participants	Single counseling session aimed at lifestyle changes can be effective at decreasing CVD risk factors. The participation of subjects with high blood pressure in the population of displaced returnees, exceeded the values for both Slavonia and Croatia census data
Ebrahimi (2014) [39]	Iran	EMRO	Cross sectional	Patients with cardiovascular and respiratory diseases who received medical services from the Center for Disaster and Emergency Medicine in Sanandaj, Iran during dust event days	March 2009—June 2010	–	A statistically significant increase in emergency admissions for cardiovascular diseases was demonstrated during dust storm episodes in Sanandaj, Iran(correlation coefficient (*r*) = 0.48, *p* < 0.05)
Huerga (2009) [40]	Liberia	Africa	Retrospective chart review	Patients of the medical and pediatric wards of Mamba Point Hospital, Monrovia, Liberia, one year after the end of the Liberian civil war	January 2005—July 2005	1,034 adult patients 1,509 children	Non- infectious diseases accounted for 56% of the adult deaths. The main causes of death were meningitis (16%), stroke (14%) and heart failure (10%).Cardiovascular diseases caused half of deaths due to non-infectious diseases: 25% stroke, 18% heart failure, and 10% severe hypertension. No cases of ischemic heart disease were identified
Hult (2010) [41]	Nigeria	Africa	Retrospective cohort	40 year old Nigerians with fetal exposure to famine in Biafra, Nigeria during the Nigerian civil war (1967–1970)	June 2009–July 2009	1,339 study participants	Fetal-infant exposure to famine was associated with elevated systolic (+ 7 mmHg; *p* < 0.001) and diastolic (+ 5 mmHg; *p* < 0.001) blood pressure, waist circumference (+ 3 cm, *p* < 0.001), increased risk of systolic hypertension (adjusted OR 2.87; 95% CI 1.90–4.34), and overweight status (OR 1.41; 95% CI 1.03–1.93) as compared to people born after the famine
Hung (2013) [42]	China	Western Pacific	Retrospective chart review	Patients treated by Hong Kong Red Cross three weeks after the 2008 Sichuan earthquake	June 2008	2,034 patient encounters	There was a high prevalence of chronic disease after the earthquake, especially hypertension. 43.4% of the 762 patients with blood pressure measurements were above the recognized criteria for hypertension
Kadojic (1999) [43]	Croatia	Europe	Cohort study	Displaced persons aged 20-60y with signs of PTSD and a history of traumatic war experience living in a displaced persons camp since 1991	–	120 displaced persons	Displaced persons in Croatia residing in camps had a significantly higher prevalence (*p* < 0.05) of hypertension, hyperlipidemia, and obesity when compared to age-matched controls in settlements adjacent to the study population not impacted by the war. Total risk for stroke was higher in the exposed group(*p* < 0.05
Kallab (2015) [44]	Country of Asylum: Lebanon Country of Origin: Syria	EMRO	Program implementation reflection	Syrian refugees and vulnerable Lebanese being treated in 8 health facilities run by Amel Association International	November 2014- May 2015	1,825 patients	Of the 1,825 patients enrolled in the program hypertension and diabetes accounted for 46% and 27% of cases respectively, with the remaining 27% of patients presenting with both diseases. Major challenges included medications shortages and cost, insecurity, patient transportation cost, and high workload for providers
Khader (2014) [45]	Country of Asylum: Jordan Country of Origin: Palestinian Territories	EMRO	Retrospective cohort study with program and outcome data collected and analyzed using E-Health	Palestine refugees living in Jordan	October 2009- June 2013	18,881 patients	50% of patients were diagnosed with both hypertension and diabetes and 50% had hypertension alone. There were significantly more patients with hypertension and diabetes (*N* = 966, 13%) who had disease- related complications than patients who had hypertension alone (*N* = 472, 6%) [OR 2.2, 95% CI 2.0–2.5]. Most common risk factors included smoking, physical inactivity, and obesity
Marjanovic (2003) [46]	Croatia	Europe	Retrospective chart review	Patients examined at Beli Manastir Health Center Department of Emergency in Baranya, Croatia post- war	November 1997 (the time of Baranya reintegration into the legal system of the Republic of Croatia after the war)—December 2001	513 stroke patients	Stroke patients presenting to the emergency department at a single site had an average of 68.4y, with an age range from 25-91y, and a near equal distribution between men and women (51.7% male). Only 50.6% of patients presented within 6 h, another 16.2% presented after 24 h. Paresis, speech impairment and vision impairment were the most common presenting symptoms. 85.8% of patients had hypertension, 27% had diabetes, 44.6% had hyperlipidemia and 46% also had cardiac disease. 38.4% of patients presenting to the hospital died
Markoglou (2005) [47]	Kosovo	Europe	Cross sectional	Patients under the care of the NATO forces who provided medical services to the civilians of Kosovo during the Yugoslav Wars	January 2000—July 2000	830 patients	30.6% patients were diagnosed with hypertension (188 female and 66 male). More than half of the patients (51.2%) had severe hypertension, 31.5% modest and 17.3% mild. Only 5.5% of patients were on regular antihypertensive treatment (9.09% men and 4.24% women). Concomitant diseases in our patients (62% of patients) were in descending order by incidence rheumatic, cardiovascular and COPD disorders. Hypertension due to increased sympathetic activity(attributed to war stress) was present in 35 patients, (13.78%, 32 – 17.02% women and 3 – 4.55% men, *p* < 0.05), and hypertension secondary to the use of NSAIDs or cortisone in 15 patients (5.91%, 8 women – 4.26% and 7 men – 10.6%, *p* > 0.1)
Mateen (2012) [48]	Country of Asylum: Jordan Country of Origin: Iraq	EMRO	Retrospective Cohort	Iraqi refugees receiving UNHCR health assistance in Jordan	January 2010-December 2010	7,642 registered Iraqi refugees	For adults 18y and older, primary hypertension was the top diagnosis(22%). Diagnoses requiring the greatest number of visits per refugee were cerebrovascular disease (average of 1.46 visits per refugee); senile cataract (1.46); glaucoma (1.44); urolithiasis (1.38); prostatic hyperplasia(1.36); and angina pectoris (1.35). Concomitant disease was common (60% has more than one diagnosis)
Miric (2001) [49]	Croatia	Europe	Retrospective chart review	Patients hospitalized in coronary care units of Clinical Hospital Split prior to, during, and following the Croatian War of Independence	1989—1997	3,454 patients	In the 3-year period preceding the war, from 1989 to 1991, 1,024 patients were hospitalized because of MI. During the 3 years of full war activities, from 1992 to 1994, there were 1,257 patients (significantly more; *p* < 0.05). And in the 3-year period after the war, from 1995 to 1997, there were 1,173 patients. Older age was a risk factor for greater morbidity and mortality, however the number of smokers was greater among patients younger than 45 years (75% vs. 51%; *p* < 0.001)
Mousa (2010) [50]	Country of Asylum: Jordan, Lebanon, Syria, West Bank/Gaza Country of Origin: Palestinian Territories	EMRO	Case series	Refugees registered by the United Nations Relief and Works Agency for Palestine Refugees in the Near East (UNRWA)	June 2007	7,762 refugees	Overall 18.7% of the screened population presented with high blood pressure (≥ 140/ ≥ 90 mmHg). People were referred for screening most commonly because of age (both sexes), followed by smoking (males) and family history (females). More females over 40 years of age were screened than men (*p* < 0.01)
Otoukesh (2012) [51]	Country of Asylum: Iran Country of Origin: Afghanistan	EMRO	Retrospective cross sectional	Afghan refugees in Iran	2005 -2010	23,152 refugees	Ischemic heart diseases constituted the fourth leading cause of referrals (10.4% of referrals). Referrals by Pashtun group were mostly for neoplasms (17%), among Uzbek group it was nephropathies (26%), and in Baluch group hematopoietic disorders (25%)
Sibai (2001) [52]	Lebanon	EMRO	Retrospective cohort study	Lebanese aged 50 years and over residing in Beirut, Lebanon in 1983	1983–1993	1,567 cases	The most important causes were non-communicable diseases, mainly circulatory disease (60%); and cancer (15%). Among circulatory diseases, ischaemic heart disease accounted for the majority of the mortality burden (68%) followed by cerebrovascular diseases (21%). In countries that lack reliable sources of mortality data, the utility of verbal autopsy can be viably extended to cohort studies for assessing causes of death
Sibai (2007) [30]	Lebanon	EMRO	Retrospective cohort study	Lebanese aged 50 years and over residing in Beirut, Lebanon	1984–1994	1,567 cases	Most important causes of death were CVD and Cancer. High adjusted risk of CVD mortality associated with being single (never-married) versus married among men and women. Outcomes were self-reported
Strong (2015) [53]	Country of Asylum: Lebanon Country of Origin: Syria	EMRO	Cross sectional	Syrian refugees over age 60 residing in Lebanon and registered with either Caritas Lebanon Migrant Center (CLMC) or the Palestinian Women’s Humanitarian Organization (PALWHO)	March 2011—March 2013	210 refugees	Older refugees reported a high burden of chronic illnesses and disabilities. Hypertension was most common (60%), followed by diabetes mellitus (47%), and heart disease (30%). The burden from these diseases was significantly higher in older Palestinians compared to older Syrians, even when controlling for the effects of sex and age (hypertension *p* < 0.001; diabetes *p* < 0.001; heart disease *p* = 0.042). Financial difficulties were given as the primary reason for not seeking care by 79% of older refugees
Sun (2013) [54]	China	Western Pacific	Cross sectional	Survivors of Wenchuan earthquake staying in a temporary shelter for more than 1 year	March–May 2009	3,230 adults	The prevalence rate of hypertension among survivors was 24.08%. Age, family history of hypertension, sleep quality, waist-to-hip ratio, BMI,and blood glucose levels are risk factors for earthquake-induced hypertension. Mental stress was not a risk factor. The rates of hypertension awareness, dosing, and control was 34.58%, 53.43% and 17.84%, respectively
Tomic (2009) [50]	Bosnia and Herzegovina	Europe	Retrospective case control	Pregnant women with hypertensive disorders and their neonates hospitalized in the Obstetric/Gynecological and Pediatric Departments of Mostar Hospital during the war and postwar period (Bosnian War 1992–1995)	January 1995—December 1999	542 pregnancies with hypertensive disorders	The prevalence of hypertensive disorders in pregnancy was higher during wartime, demonstrated by a drop in prevalence during the five years after the war, with the highest prevalence occurring at 8.7% during the first year after the war. Those in the study group had higher odds of placental abruption, cesarean delivery, preterm birth, fetal growth restriction, and fetal death. Those in the study group with hypertensive pregnancy disorders had a lower number of prenatal care visits than controls (*p* < 0.001)
Vasilj (2006) [55]	Bosnia and Herzegovina	Europe	Retrospective chart review	Patients who suffered from the acute coronary syndrome in western Herzegovina pre, during, and post-war (Bosnian War 1992–1995)	1987–2001	2,022 patients	There was a higher prevalence of ACS presentations both during (*n* = 665, *p* < 0.0005) and after the war (*n* = 843, *p* < 0.0005), as compared to prior to the war (*n* = 365) in both sexes
Vukovic (2005) [56]	Serbia	Europe	Retrospective chart review	Patients with ischemic heart disease who were admitted to the Cardiac policlinic for a control check-up immediately after the suspension of air raids	June 1999	75 patients	The severity of angina pains and nitroglycerin pill usage was associated with timing of air raids, increasing during the first week and initial week after raids when compared to the week before raids
Yusef (2000) [22]	Country of Asylum: Lebanon Country of Origin: Palestinian Territories	EMRO	Cross-sectional	Diabetic and hypertensive patients attending UNRWA primary health care facilities in Lebanon	1997	2,202 records	Presence of both diabetes and hypertension increased the risk for late-stage complications. The major complication was cardiovascular disease followed by retinopathy. Only 18.2% of diabetic patients and 17.7% of diabetic patients with hypertension were managed by lifestyle modification. Medication shortages may drive medication choices for hypertension
Zubaid (2006) [57]	Kuwait	EMRO	Retrospective chart review	Catchment area of Mubarak Al Kabeer Hospital	March 2003	1 Missile Attack Period (MAP) and 4 control periods	Missile attacks were associated with an increase in the incidence of AMI. The number of admissions for AMI was highest during MAP, 21 cases compared to 14–16 cases in the four control periods, with a trend towards increase during MAP (incidence rate ratio = 1.59; 95% CI 0.95 to 2.66, *p* < 0.07).The number of admissions for AMI during the first 5 days of MAP was significantly higher compared to the first 5 days of the four control periods (incidence rate ratio = 2.43; 95% CI 1.23 to 4.26,*p* < 0.01)

**Table 2:** Characteristics of included publications by disease type: Cancer

	**Country/** **Territory of Interest**	**WHO region**	**Type of study**	**Target Population**	**Years of observation**	**Number of study participants**	**Major findings**
Huynh (2004) [58]	Vietnam	Western Pacific	Case control	Vietnamese women hospitalized with cervical cancer	June 1996—September 1996	145 women in southern Vietnam and 80 women in northern Vietnam	The development of invasive cervical cancer was significantly associated with military service by husbands during the Second Indochinese War and with parity status. Geographic and temporal variation in cervical cancer rates among Vietnamese women was associated with the movement of soldiers
Khan (1997) [59]	Country of Asylum: Pakistan Country of Origin: Afghanistan	EMRO	Cross sectional	Patients from North West Pakistan and Afghan refugees attending the Institute of Radiotherapy and Nuclear Medicine, Peshwar	1990—1994	13,359 patients 2988 were Afghan refugees 10,371 were adults from North West of Pakistan	In male Afghan refugees, esophageal cancer represented 16.6% of the cases, compared to only 4.6% of the cases in Pakistani residents. Both Pakistani and Afghani refugee women experienced breast cancer as the most common cancer
Li (2012) [60]	China	Western Pacific	Retrospective cohort	Birth cohorts who were exposed to the 1959–1961 Chinese famine	1970–2009	Population of Zhaoyuan county during the 1970–1974 death survey and 2,830,866 during the 2005–2009 death survey	The Zhaoyuan population, which experienced long-term nutritional deficiencies from childhood to adolescence, had increased risk for stomach cancer 15 to 20 years after the 1959–1961 Chinese famine. The birth cohorts who were exposed to famine or experienced malnutrition had higher stomach cancer mortality rates in later life than the birth cohorts not exposed to malnutrition
Marom (2014) [61]	Philippines	Western Pacific	Case series	Patients presenting with head and neck (H&N) tumors to a field hospital in the ‘sub-acute’ period following a typhoon	November 2013	1844 adult patients examined, 85 (5%) presented with H&N tumors	In a relief mission, despite the lack of clinical and pathological staging and questionable continuity of care, surgical interventions can be considered for therapeutic, palliative and diagnostic purposes
McKenzie (2015) [62]	Country of Asylum: Jordan Country of Origin: Iraq, Syria	EMRO	Retrospective cohort	UNHCR registered refugees (Iraqi/Syrian) in Jordan	2012—2013	223 refugees	Brain tumors accounted for 13% (*n* = 29) of neuropsychiatric applications, and was the most expensive neuropsychiatric diagnosis overall and per applicant. The ECC denied six applications for reasons of eligibility, cost, and/or prognosis. Of the 20 approved applications, 15% (*n* = 3) were approved for less than the requested amount, receiving on average 39% of requested funds
Milojkovic (2005) [63]	Croatia	Europe	Retrospective cohort	Patients with corpus uteri and cervix uteri cancer and ovarian cancer treated in the Clinical Hospital Osijek	1984 -2002	1455 patients treated for gynecological cancer were analyzed	Gynecologic cancer incidence according to age shows an increase tendency of cervical cancer in younger women in the post war period. The incidence of corpus cancer and ovary has not changed in the observed periods
Otoukesh (2012) [51]	Country of Asylum: Iran Country of Origin: Afghanistan	EMRO	Cross sectional	Afghan refugees in Iran	2005–2010	23,152 refugees	Neoplasms represented 17% of referrals among Pashtun group
Shamseddine (2004) [64]	Lebanon	EMRO	Ecological study	Lebanese cancer patients following the 1975 -1990 Lebanese Civil War	1998	4388 cases	Among males, the most frequently reported cancer was bladder (18.5%), followed by prostate (14.2%), and lung cancer (14.1%). In sharp contrast to countries worldwide, bladder cancer was notably high, in particular among males. Among females, breast cancer alone constituted around one third of the total cancer caseload in the country. This was followed by colon cancer (5.8%), and cancer of the corpus uteri (4.8%). The predominance of smoking related cancers highlights the importance of primary preventive strategies aimed at reducing smoking prevalence in Lebanon
Sibai (2001) [52]	Lebanon	EMRO	Retrospective cohort	Retrospective cohort study Lebanese aged 50 years and over residing in Beirut, Lebanon in 1983–1993 during the Lebanese Civil War	1983–1993	1567 cases	In both sexes, the leading causes of death were non-communicable, mainly circulatory diseases (60%) and cancer (15%)
Telarovic (2006) [65]	Croatia	Europe	Cross sectional	Patients with CNS tumors admitted to the Department of Neurology of Pula General Hospital, Croatia during wartime	January 1986-December 2000	364 patients	There was a statistically significant increase of incidence rate ratios (IRR) of CNS tumors in war period versus the periods before and after war..Higher proportion of metastatic tumors than expected per the authors literature review. Authors relate to stress and PTSD

**Table 3:** Characteristics of included publications by disease type: Chronic Respiratory Disease

	**Country/Territory of Interest**	**WHO region**	**Type of study**	**Target Population**	**Years of observation**	**Number of study participants**	**Major findings**
Abul (2001) [66]	Kuwait	EMRO	Retrospective chart review	Patients admitted with asthma in Kuwait	2001	12,113 asthma patients during the pre-Gulf War period compared with 9,771 patients during the post-Gulf War period	No significant difference between hospitalization or death rates pre and post Gulf War
Bijani (2002) [67]	Iran	EMRO	Retrospective chart review	Patients exposed to chemical weapons in northern Iran	1994—1998	220 patients	Obstructive lung disease was a common finding amongst patients exposed to chemical weapons in Iran
Ebrahimi (2014) [68]	Iran	EMRO	Retrospective chart review	Patients with respiratory or cardiac diseases in Sanandaj, Iran	March 2009—June 2010	–	Cardiac disease, but not respiratory disease, was significantly correlated with dust storm events
El-Sharif (2002) [69]	West Bank/Palestinian Territories	EMRO	Retrospective chart review	Schoolchildren in Ramallah District, Palestine	Autumn of 2000	3,382 children	Children from refugee camps appear to be at higher risk of asthma than children from neighboring villages or cities. Multivariate logistic regression confirmed that the estimated risk of having wheezing in the previous 12 months was higher for those residing in refugee camps than those living in neighboring villages and cities
Forouzan (2014) [70]	Iran	EMRO	Prospective observational	Patients presenting with asthma or bronchospasm in western Iran	Nov-13	2000 patients	Many patients presented with bronchospasm after a thunderstorm
Hung (2013) [42]	China	Western Pacific	Cross-sectional chart review	Patients presenting during 19 days following the Sichuan earthquake	Jun-08	2,034 patients	Musculoskeletal, respiratory, and GI problems were the top 3 areas and > 43% of patients had BP in HTN range
Kunii (2002) [71]	Indonesia	South-East Asia	Cross sectional	Patients exposed to air pollution in the “haze disaster” in Indonesia	September 1997 -October 1997	543 subjects	Patients had increased respiratory issues after a large forest fire disaster, especially the elderly. Wearing a high quality face mask was protective (vs handkerchief or simple surgical mask)
Lari (2014) [72]	Iran	EMRO	Cross sectional	Patients exposed to sulphur mustard gas	March 2010- April 2011	82 patients	The COPD Assessment Test (CAT) was found to be a valid tool for assessment of health related quality of life in chemical warfare patients with COPD
Mirsadraee (2011) [73]	Iran	EMRO	Retrospective Cohort	Patients whose parents were exposed to chemical warfare	–	409 children	The prevalence of asthma was not significantly different in the offspring of chemical warfare victims
Molla (2014) [74]	Bangladesh	South-East Asia	Cross sectional	Children 5 years of age in Dhaka with diarrhea and asthma	September 2012 -November 2012	410 households	The DALYs lost due to asthma and diarrhea were significantly different amongst the climate refugee community than a non refugee group
Naumova (2007) [75]	Ecuador	Americas	Cross sectional chart review	ED patients after a volcanic eruption in Quito, Ecuador	January 2000 -December 2000	5,169 patients	Rate of ED visits for respiratory conditions significantly increased in 3 weeks after eruption. Rates of asthma and asthma related diagnosis double during volcano “fumarolic activity”. 345 excess ED visits in 4 weeks
Guha-Sapir(2007) [76]	Indonesia	South East Asia	Cross sectional	Patients attending an International Committee of the Red Cross(ICRC) field hospital in Aceh, Indonesia, established immediately after the tsunami in 2004	2 January 15, 2004- January 31 2,004,005–2010	1,188 study participants	Post tsunami, respiratory diseases were one of the most commonly recorded conditions (21.0%) and included acute asthma exacerbations
Redwood-Campbell (2006) [77]	Indonesia	South-East Asia	Cross Sectional	Patients registering in the ICRC field hospital in Banda Aceh after the tsunami	Mar-05	271 patients	12% of the problems seen in the clinic 9 weeks after the tsunami were still directly related to the tsunami. Majority of patients were male, the problems were urologic, digestive, respiratory and musculoskeletal in that order. 24% had 4 or more depression/PTSD symptoms
Wright (2010) [78]	Kuwait	EMRO	Cross sectional	Patients in Kuwait following the Iraqi invasion	December 2003—January 2005	5028 subjects	Study suggested that those who reported highest stress exposure in the invasion were more than twice as likely to report asthma. Suggestive of correlation between war trauma and asthma

**Table 4:** Characteristics of included publications: Diabetes Mellitus

	**Country/** **Territory of Interest**	**WHO region**	**Type of study**	**Target Population**	**Years of observation**	**Number of study participants**	**Major findings**
Abukhdeir (2013) [32]	Palestinian Territories: West Bank/Gaza	EMRO	Cross sectional	Palestinian households in the West Bank and Gaza Strip	May 2004—July 2004	4456 households in the West Bank and 2118 in the Gaza Strip	Being a refugee was a significant risk factor for diabetes and CVD while being married/engaged or divorced/separated/ widowed was a risk factor for diabetes and hypertension. Non-refugees were 33% less likely to have diabetes and 46% less likely to have CVD than refugees. Gender was a risk factor for hypertension with females being 60% more likely to have hypertension than males
Ahmad (2015) [33]	Syria	EMRO	Situational analysis using document analysis, key informant interviews, and direct clinic observation	Syrian national health system	October 2009 -August 2010	53 semi-structured interviews	The rebuilding of a post-conflict heath care system in Syria may benefit from insights into the structural problems of the pre-crisis system. Weaknesses that existed before the crisis are compounded by the current conflict. The authors suggest an over reliance on secondary and tertiary care for DM patients with withdrawal of the Syrian government from the public health clinics, which led to escalating healthcare costs and fostered increasingly unequal access
Alabed (2014) [79]	Country of Asylum: Syria Country of Origin: Palestinian Territories	EMRO	Cross sectional	Palestinian refugees living in Damascus attending three UNRWA health clinics	August 2008—September 2008	154 DM patients	UNRWA clinic inspections highlighted shortages in drug stocks with 47.3% of patients reporting problems accessing prescribed medications and 67.7% reporting having to buy medications at their own expense at least once since their diagnosis. Patients’ knowledge of their condition was limited, Patients were generally unaware of the importance of good glucose control and disease management. Women were more likely to attend the clinic than men, with 71% of patients being female
Ali-Shtayeh (2012) [80]	Palestinian Territories: West Bank	EMRO	Cross sectional	Patients attending outpatient departments at West Bank Governmental Hospitals in 7 towns in the Palestinian territories (Jenin, Nablus, Tulkarm, Qalqilia, Tubas, Ramalla, and Hebron)	August 2010—May 2011	1,883 DM patients	While all patients using complementary and alternative medicine (CAM) were additionally using conventional therapies, the use of CAM differed significantly between residents of refugee camps versus residents of urban or rural areas (*p* = 0.034). More residents in a refugee camp reported using CAM vs. not using CAM as compared to those who reported living in a village or city. Most CAM users were above 40 years old, predominantly female, and residents of refugee camps and rural areas
AlKasseh (2013) [81]	Palestinian Territories: Gaza	EMRO	Retrospective case control	Refugee women attending the UNRWA postnatal clinics in Gaza	March 2011—June 2011	189 postnatal GDM women with 189 matched controls by age and place of residency	A history of miscarriage more than once, being overweight before pregnancy, history of stillbirth, history of caesarean birth and positive family history of diabetes mellitus were strongly correlated with developing gestational diabetes(GDM). WHO criteria for screening for GDM remain a good instrument to identify GDM in refugee populations in war-torn countries (like the Gaza Strip)
An (2014) [82]	China	Western Pacific	Retrospective cohort	1976 Tangshan Earthquake survivors, aged 37–60, without severe liver disease, trauma surgery, secondary diabetes, or diagnosed mental disease	September 2013—December 2013	1030 exposed subjects	The incidences of impaired fasting glucose and DM for earthquake survivors were significantly higher than that for the control group. There was a higher diabetes incidence in those who had lost relatives than those who had not lost relatives, however, this effect was only statistically significant in women earthquake survivors
Armenian (1998) [83]	Armenia	Europe	Retrospective cohort	Employees of the Armenian Ministry of Health and their immediate families who survived the 1988 Earthquake in Armenia	1990–1992	35,043 persons (7,721 employees who had survived the disaster and their family members)	Longer term increased rates of DM morbidity following an earthquake are related in a dose–response type relationship to the intensity of exposure to disaster. Bereavement, injuries in the family, and material loss, act as independent predictors of long term adverse physical illness including for DM
Balabanova (2009) [84]	Georgia	Europe	Rapid appraisal process with snowball sampling	Georgian health system evaluation	March—April 2006	36 interviews	Essential inputs for diabetes care are in place (free insulin, training for primary care physicians, financed package of care), but constraints within the system hamper the delivery of accessible and affordable care. The scope of work of primary care practitioners is limited and they rarely diagnose and manage diabetes, which instead takes place in the context of a hospital admission and tertiary-level endocrinologists. Obtaining syringes, supplies and hypoglycemic drugs and self-monitoring equipment remains difficult and leads to a cost driven shift toward insulin for diabetic management
Ben Romdhane (2015) [85]	Tunisia	EMRO	Situational analysis	Tunisian national health system	2010	12 key informants were interviewed and eight documents were reviewed	Weaknesses that existed before the 2011 Revolution(Arab Spring) were compounded during the revolution. This study was conducted prior to political conflict but written post-conflict. Growth of the private sector fostered unequal access by socioeconomic status and reduced coordination and preparedness of the health system
Besancon (2015) [86]	Mali	Africa	Case study	Mali diabetic population following a March 2012 Coup in Bamako	Spring 2012 following the March 2012 coup	–	Diabetics are a vulnerable population in humanitarian crisis due to their continuous need for health care and medicines and the financial burden this may place on them. The authors propose that in an emergency setting there is not one single diabetes population that should be considered in planning humanitarian responses, but multiple, each with unique needs. These sub-populations include people still in active conflict regions, IDPs, refugees, and the population which houses IDPs
Ebling (2007) [87]	Croatia	Europe	Multipart study including both a retrospective cohort study and an uncontrolled before-after study	Refugee-returnees of the 1991–1992 war operations in Eastern Slavonia from Osjek-Baranga County, Croatia	2003	retrospective cohort study: 589 participants uncontrolled before-after study 202 participants	The participation of subjects with DM in the population of refugee-returnees despite similar demographic indicators, exceeded values for both Slavonia and Croatia. Extremely high participation of patients with diabetes was noted(10.5%), despite a lower proportion of aged people over 65 among returnees
Eljedi (2006) [88]	Palestinian Territories: Gaza	EMRO	Cross sectional	Patients with DM residing in refugee camps in Gaza Strip	November 2003—December 2004	197 DM patients	Using the World Health Organization Quality of Life questionnaire (WHOQOL-BREF) four domains–including physical health, psychological, social relations, and environment – were strongly reduced in diabetic patients as compared to controls, with stronger effects in physical health (36.7 vs. 75.9 points of the 0–100 score) and psychological domains (34.8 vs. 70.0) and weaker effects in social relationships (52.4 vs. 71.4) and environment domains (23.4 vs. 36.2). The impact of diabetes on health-related quality of life (HRQOL). was especially severe among females and older subjects (above 50 years)
Gilder (2014) [89]	Country of Asylum: Thailand Country of Origin: Myanmar	South-East Asia	Cross sectional	Women attending the antenatal care (ANC) clinic in Maela refugee camp on the Thai–Myanmar border	July 2011—March 2012	228 women	The prevalence of GDM is lower in this population compared with other populations, but still complicates 10% of pregnancies. Despite the weight of evidence for the benefits of early diagnosis and treatment of GDM, the absence of a simple, inexpensive and applicable screening method remains a major barrier to GDM screening programs in refugee camps and other resource-poor settings
Habtu (1999) [90]	Ethiopia	Africa	Cross Sectional	Insulin treated diabetic patients from the Diabetic Clinic at the Mekelle Hospital in rural Tigray, Northern Ethiopia- the center of the severe Ethiopian famine of the mid-1980s	Six month period in 1997	100 patients	The correct prescribed dose of insulin was only being taken by 50% of patients and the correct syringe by only 12%. Insulin treatment had been interrupted in 48% of cases due to lack of supply. Low BMI(mean of 15.8), young age, and resistance to diabetic ketoacidosis(DKA) amongst study participants were consistent with previous descriptions of malnutrition related diabetes mellitus(MRDM)
Hult (2010) [42]	Nigeria	Africa	Retrospective Cohort	40 year old Nigerians with fetal exposure to famine in Biafra, Nigeria during the Nigerian civil war (1967–1970)	June 2009–July 2009	1,339 study participants	Fetal and infant undernutrition was associated with significantly increased risk of impaired glucose tolerance in 40 year old Nigerians. However, early childhood exposure was not associated with increased risk
Kallab (2015) [44]	Country of Asylum: Lebanon Country of Origin: Syria	EMRO	Program implementation reflection	Syrian refugees and vulnerable Lebanese host communities over the age of 40	November 2014- May 2015	1825 patients	DM accounted for 54% of patient cases, with 27% of patients affected by both DM and HTN. Principal barriers to providing diabetic management in active conflict included insecurity, the fluid movement of refugees, limited opening hours of the centers, transportation costs, and medication shortages
Karrouri (2014) [91]	Country of Asylum: Tunisia Country of Origin: Libya	EMRO	Case report	Case of a 10-year-old Libyan boy	–	One patient	Report of a 10 year old without personal or familial diabetes mellitus history who developed type 1 diabetes appeared immediately following severe psychological trauma
Khader (2012) [82]	Country of Asylum: Jordan Country of Origin: Palestinian Territories	EMRO	Retrospective cohort	Persons with DM at Nuzha PHC Clinic	October 2009- March 2012	2,851 patients	A directly observed therapy(DOTS) cohort monitoring system can be successfully adapted and used to monitor and report on Palestinian refugees with DM in Jordan. A sizeable proportion of DM patients of the clinic failed to have postprandial blood glucose measurements, and BP measurements in those with comorbid HTN
Khader (2013) [77]	Country of Asylum: Jordan Country of Origin: Palestinian Territories	EMRO	Retrospective cohort	Palestine refugees living in Jordan	October 2009- June 2013	12,549 total patients	High burden of disease due to DM amongst Palestinian refugees at UNRWA primary health care clinics in Jordan. Cohort analysis using e-Health is a successful tool for to assess management and follow-up of DM patients. Complications, including myocardial infarction and end-stage renal disease were significantly more common in males. Females were more likely to be obese
Khader (2014) [69]	Country of Asylum: Jordan Country of Origin: Palestinian Territories	EMRO	Retrospective cohort	Palestinian refugees living in Jordan with DM attending Nunzha Clinic	2012	2,974 DM patients	E-Health systems are useful for monitoring patients, since over half who miss their quarterly appointment fail to return. Suggests a need for monitoring and active follow-up
Khader (2014) [45]	Country of Asylum: Jordan Country of Origin: Palestinian Territories	EMRO	Retrospective cohort	Palestinian refugees living in Jordan with DM attending Nunzha Clinic	2010–2013	119 DM patients	E-health systems are useful for monitoring patients. An increasing number of patients had complications despite no change in obesity rates indicating places where more resources may be useful
Li (2010) [72]	China	Western Pacific	Retrospective cohort	Rural Chinese exposed to the Chinese famine(1959–1961) during fetal life and early childhood	2002	7,874 rural Chinese	In severely affected famine areas, fetal-exposed adults had an increased risk of hyperglycemia compared with nonexposed subjects. Differences were not significant for the early and mid childhood–exposed cohorts. This association appears to be exacerbated by a nutritionally rich environment in later life
Lumey (2015) [92]	Ukraine	Europe	Retrospective cohort	Individuals exposed to the man-made Ukrainian famine of 1932–33 during prenatal development compared with all patients with type 2 diabetes diagnosed at age 40 years or older in the Ukraine national diabetes register 2000–08	2000–2008	43,150 patients with diabetes and 1,421,024 controls	Demonstrates a dose–response relationship between famine severity during prenatal development and odds of type 2 diabetes in later life. The associations between type 2 diabetes and famine around the time of birth were similar in men and women
Mansour (2008) [93]	Iraq	EMRO	Cross sectional	Diabetic patients in an outpatient clinic in Al-Faiha general hospital in Basrah, South Iraq	January 2007—December 2007	3,522 diabetic patients	The most common reasons for poor glycemic control(HBA1C > 7%) listed by patients were drug shortages and drugs and/or laboratory expense(over 50%). 30% of diabetic patient with poor glycemic control believed that their poor glycemic control is due to migration after the war
Mateen (2012) [48]	Country of Asylum: Jordan Country of Origin: Iraq	EMRO	Cross sectional	Iraqi refugees receiving health assistance in Jordan as recorded by a UNHCR database	January 2010-December 2010	7642 Iraqi refugees	11% of refugees presented with type 2 DM. For all refugees the largest number of visits were for essential hypertension (2067 visits); visual disturbances (1129); type II diabetes mellitus (1021)
Mousa (2010) [50]	Country of Asylum: Jordan, Syria, Lebanon, Gaza, West Bank Country of Origin: Palestinian Territories	EMRO	Cross sectional	UNRWA registered Palestinian refugees attending UNRWA clinics	June 2007	7,762 refugees	Overall 9.8% of screened refugees had random blood glucose values ≥ 126 mg/dL. Being older than 40 years, obese or with a positive family history of diabetes or cardiovascular disease increased the risk of presenting with hyperglycemia 3.5, 1.6 and 1.2 times respectively. Variations were statistically significant between UNRWA locations and between the sexes. Significant variations were found between fields for females (χ2 = 112.6, *P* < 0.01) and for males (χ2 = 39.2, *P* < 0.01), with the highest proportion of cases diagnosed in the Occupied Palestinian Territories and the lowest in Jordan and Syria
Ramachandran (2006) [94]	India	South-East Asia	Retrospective cohort	Tsunami affected population of Chennai(Madras) in Southern India	April 2005- June 2005	1,184 tsunami affected subjects, 1,176 controls	Undetected diabetes and impaired glucose tolerance were higher in the tsunami-hit area as compared to controls. Diabetes prevalence was found to be similar in the tsunami affected population and control. Women of both the control and the tsunami affected population had both a higher stress score(using the Harvard trauma questionnaire) than men with a significantly higher stress score in women affected by the tsunami, as well as a higher prevalence of impaired glucose tolerance in the tsunami hit area
Read (2015) [67]	The Philippines	Western Pacific	Cross sectional	Patients treated by an Australian Government deployed surgical team in a field hospital in the city of Tacloban for 4 weeks after Typhoon Haiyan	November 2013	131 persons	Sepsis from foot injuries in diabetic patients constituted an unexpected majority of the workload of a foreign collaborative surgical medical team in Tacloban in the aftermath of Typhoon Haiyan
Sengul (2004) [95]	Turkey	Europe	Prospective cohort	Type 1 Diabetic Survivors of the 1999 Marmara Earthquake	1998–2000	88 subjects	HbA1c levels and insulin requirements significantly increased at the 3rd month post earthquake however only increased insulin requirement continued to be significantly increased, one year post earthquake. No significant difference was identified between HbA1c levels pre earthquake and post 1 year earthquake. Results indicated that the Marmara earthquake affected glycemic control of people with type 1 diabetes in the short term but its negative impact did not continue in long term
Sofeh (2004) [94]	Country of Asylum: Pakistan Country of Origin: Afghanistan	EMRO	Cross sectional	Adult Afghan Refugees attending Red Cross health care facilities in Peshawar, Pakistan	.	456 patients	The frequency of non-insulin dependent DM was found to be 55.9% amongst Afghan refugees in Peshawar during a two year study period. 17.25% of diabetics had concomitant hyperlipidemia. Gender was not identified as a risk factor for higher fasting blood glucose levels
Strong (2015) [53]	Country of Asylum: Lebanon Country of Origin: Syria	EMRO	Cross sectional	Syrian refugees over age 60 residing in Lebanon and registered with either Caritas Lebanon Migrant Center (CLMC) or the Palestinian Women’s Humanitarian Organization (PALWHO)	March 2011—March 2013	210 refugees	47% of older refugees had DM. The number of days older refugees reporting eating bread only and nothing else corresponded to their reported financial status. Financial difficulties were given as the primary reason for not seeking care by 79% of older refugees with only 1.5% stating they had no difficulties in obtaining care when needed
Wagner (2016) [96]	Cambodia	Western Pacific	Uncontrolled before and after	Unpaid Cambodian village health guide volunteers were trained in DM prevention teaching behaviors	.	185 guides were trained to instruct at 10 health centers	Knowledge of community health workers on DM prevention techniques increased significantly from pre-test to posttest after 6 months of follow-up. 159 guides (85%) completed at least one monthly checklist
Yaghi (2012) [97]	Lebanon	EMRO	Cross sectional	Cases of amputations in Lebanon	January 2007-December 2007	661 amputations	Diabetes and vascular indications were not only more common than trauma-related amputation, but both were associated with more major surgery and longer hospital stay including conflict afflicted southern Lebanon where trauma, diabetes and vascular disease amputations all occurred at more than twice the national rate
Yusef (2000)[22]	Country of Asylum: Lebanon Country of Origin: Palestinian Territories	EMRO	Cross sectional	Diabetic and hypertensive patients attending UNRWA primary health care facilities in Lebanon	1997	2,202 records	Presence of both DM and HTN increased the risk for late-stage complications. Only 18.2% of diabetic patients and only 17.7% of DM patients with HTN were managed by lifestyle modification. About 50% of type 2 and 66% of type 1 patients who were on insulin were well controlled

**Table 5:** Characteristics of included publications by disease type: Other Non-Communicable Diseases

	**Country/Territory of Interest**	**WHO region**	**Type of study**	**Target Population**	**Years of observation**	**Number of study participants**	**Major findings**
Amini (2010) [98]	Iran	EMRO	Cross- sectional	Iranian war victims blinded in both eyes	2007	250 conference attendees	Quality of Life (QOL) scores in blind war victims decreased with increasing age and additional medical comorbidities
Armenian (1998) [83]	Armenia	European	Prospective, nested case–control	Survivors of the 1988 Earthquake in Armenia	1988–1992	35,043 employees of the Armenian Ministry of Health and their immediate families	During a 4-year follow-up period, the highest number of deaths from all causes (including heart disease) occurred within the first 6 months following the earthquake; associated with extent of disaster-related damage and losses
Chan (2010) [99]	Pakistan	EMRO	Cross sectional	Face-to-face, household-based survey conducted 4 months after the 2005 Kashmir, Pakistan earthquake in internally displaced camps near Muzafarabad city	February 2006 4 months post-earthquake	167 households	Although the proportion of the population with chronic conditions was similar across these studied camps, 85% of residents in the smallest unofficial camp had no available drugs to manage their chronic medical conditions as compared with their counterparts residing in larger rural unofficial (40%) and official camps (25%)
Chan (2009) [92]	Pakistan	EMRO	Comparative descriptive study	Patients ≥ 45 years who attended two different types of post-earthquake relief clinics during a 17-day field health needs assessment in response to the 2005 Kashmir earthquake	February 2006 4 months post-earthquake	30,000 patients in a rural site, and 382 IDPs in a urban site	The greatest gap in health services post-earthquake in both sites was non-communicable disease management. Clinical records reviewed in all study locations showed a systematic absence of documentation of common NCDs. In rural areas, older women were less likely to receive medical services while older men were less likely to access psychological services in both sites. During days when solely male doctors provided clinical services in the rural site, medical services utilization decreased by 30%
Hung (2013) [42]	China	Western Pacific	Cross-sectional chart review	Patients presenting during a 19 day period three weeks following the Sichuan earthquake	Jun-08	2,034 patients	Musculoskeletal, respiratory, and GI systems were top 3 problems and > 43% of patients met hypertension criteria
Khateri (2003) [100]	Iran	EMRO	Cross-sectional retrospective survey	Patients exposed to chemical weapons in Iran during the Iran-Iraq War (1980–1988)	1997–2000	34,000 subjects	Lesions of the lungs (42.5%), eyes (39.3%), and skin (24.5%) were the most common sites of involvement among mustard agent exposure survivors
Leeuw (2014) [101]	Country of Asylum: Jordan, Lebanon Country of Origin: Syria	EMRO	Cross-sectional survey	Syrian refugee households in Jordan and Lebanon	2013	3,202 refugees	Impairments found in 22% of refugees and disproportionately affecting those over 60 years of age (70% with at least 1 impairment)
Li (2011) [102]	China	Western Pacific	Cross-sectional survey	Adults exposed to severe famine in utero or as children	2002	7,874 adults	Adults exposed to severe famine while in utero or early childhood had increased risk of metabolic syndrome
Mateen (2012) [31]	Country of Asylum: Jordan Country of Origin: Iraq	EMRO	Prospective observational	Iraqi refugees seeking health care in Jordan	2010	7642 patients	Chronic diseases like hypertension (22%) and diabetes (11%) were common and the most common reason for visit was respiratory illness (11%)
Mateen (2012) [103]	19 countries	Africa, EMRO, South East Asia	Retrospective chart review	Refugees in camp settings globally	2008–2011	58,598 visits	Chronic, noncommunicable diseases like epilepsy and cerebrovascular disease far exceeded (> 98%) those for neurologic infectious diseases
McKenzie (2015) [62]	Country of Asylum:Jordan Country of Origin: Syria, Iraq	EMRO	Retrospective cohort	Syrian & Iraqi refugees applying for emergency or exceptional medical care	2012–2013	223 refugees	Neuropsychiatric applications accounted for 11% of all Exceptional Care Committee applications and 2/3 of neuropsychiatric cases were for emergency care
Otoukesh (2012) [51]	Country of Asylum: Iran Country of Origin: Afghanistan	EMRO	Cross-sectional	Afghan refugees in Iran	2005–2010	23,152 refugees	The most common health referral for those aged 15–59 years was ophthalmic diseases in females and nephropathies in males. In those aged 60 + it was ophthalmic diseases for both sexes
Redwood-Campbell (2006) [77]	Indonesia	South East Asia	Prospective observational	Patients registering in the ICRC field hospital in Banda Aceh 9 weeks after the tsunami	Mar-05	271 patients	12% of clinic visits were directly related to the tsunami. The most common medical complaints were urological (19%), digestive (16%), respiratory (12%), and musculoskeletal (12%). 24% of patients had 4 or more depression/PTSD symptoms
Sibai (2001) [52]	Lebanon	EMRO	Cross-sectional	Representative cohort of men and women completing a health survey in Beirut, Lebanon during wartime	1983–1993	1567 subjects	Total mortality rates were estimated at 33.7 and 25.2/1000 person years among men and women respectively. Leading cause of death was circulatory disease (60%) and cancer (15%) for both sexes
Strong (2015) [53]	Country of Asylum: Lebanon Country of Origin: Syria, Palestine	EMRO	Cross Sectional	Refugees over age 60 receiving assistance from social workers	2011–2013	210 refugees	Most older refugees reported at least one non-communicable disease: hypertension (60%), diabetes (47%), heart disease (30%). 74% indicated at least some dependency on humanitarian assistance

**Table 6:** Characteristics of included publications by region: Africa

	**Country/Territory of Interest**	**Target Population**	**Type of Study**	**NCD Studied**	**Years of Observation**	**Number of study participants**	**Major Findings**
Besancon (2015) [86]	Mali	Mali diabetic population following a March 2012 Coup in Bamako	Case Study	Diabetes	Spring 2012 following the March 2012 coup	–	Diabetics are a vulnerable population in humanitarian crisis due to their continuous need for health care and medicines and the financial burden this may place on them. In an emergency setting sub-populations of diabetics must be taken into account for humanitarian response planning; including people still in active conflict regions, IDPs, refugees, and the host population which houses IDPs
Habtu (1999) [90]	Ethiopia	Insulin treated diabetic patients from the Diabetic Clinic at the Mekelle Hospital in rural Tigray, Northern Ethiopia- the center of the severe Ethiopian famine of the mid-1980s	Cross-sectional	Diabetes	Six month period in 1997	100 patients	The correct prescribed dose of insulin was only being administered in 50% of DM patients in rural Tigray, Ethiopia and the correct syringe by only 12% of patients. Insulin treatment had been interrupted in 48% of cases due to lack of supply. Low BMI(mean of 15.8), young age, and resistance to diabetic ketoacidosis(DKA) amongst study participants were consistent with previous descriptions of malnutrition related diabetes mellitus(MRDM)
Huerga (2009) [97]	Liberia	Patients of the medical and pediatric wards of Mamba Point Hospital, Monrovia, Liberia, one year after the end of the Liberian civil war	Cross-sectional	Multiple NCDs including CVD (stroke, CHF, and HTN)	January 2005—July 2005	1,034 adult patients 1,509 children	Of 1034 adult hospitalized patients in post-war Liberia, 529 (51%) were diagnosed with a noninfectious disease. Among the 241 deaths recorded, the cause was non-infectious disease in 134 (56%) patients. The fatality rate for infectious diseases (19.7%; 92 deaths/465 cases) was lower (*P* = 0.04) than for non-infectious diseases (25.3%; 134 deaths/529 cases). Cardiovascular diseases caused half of deaths due to non-infectious diseases: 25% stroke, 18% heart failure and 10% severe hypertension. No cases of ischemic heart disease were identified Among hospitalized children, 229 (15%) were diagnosed with a noninfectious disease. NCDs represented 34% of all deaths. The fatality rate for infectious diseases (18.6%; 197 deaths/1189 cases) was lower (*P* < 0.01) than for non-infectious diseases (28.8%; 66 deaths/229 cases)
Hult (2010) [42]	Nigeria	40 year old Nigerians with fetal exposure to famine in Biafra, Nigeria during the Nigerian civil war (1967–1970)	Retrospective cohort	Diabetes and HTN	June 2009–July 2009	1,339 study participants	Fetal and infant undernutrition was associated with significantly increased risk of hypertension(adjusted OR 2.87; 95% CI 1.90–4.34), and impaired glucose tolerance (OR 1.65; 95% CI 1.02–2.69) in 40 year old Nigerians. However, early childhood exposure was not associated with increased risk

**Table 7:** Characteristics of included publications by region: Region of the Americas

	**Country/Territory of Interest**	**Target Population**	**Type of Study**	**NCD Studied**	**Years of Observation**	**Number of study participants**	**Major Findings**
Daniels (2009) [104]	Peru	Displaced as well as nondisplaced populations in rural to urban settings	Stratified cluster survey	Multiple NCDs	2007 (six months post-earthquake)	672 households	Displaced populations sought care more, people with injury or NCD sought care more. People who did not seek care cited cost as a barrier
Naumova(2007) [75]	Ecuador	Pediatric ER visits with acute upper and lower respiratory conditions and asthma related conditions	Retrospective review of medical records	Chronic respiratory disease including asthma	January – 2000 December 2000	5,169 emergency department records	Rates of ED visits for pediatric patients increased significantly during period of volcanic activity. Youngest patients (4 and under) were most affected

**Table 8:** Characteristics of included publications by region: Eastern Mediterranean Region

	**Country/** **Territory of Interest**	**Target Population**	**Type of study**	**NCD studied**	**Years of observation**	**Number of study participants**	**Major findings**
Abukhdeir (2013) [32]	Palestinian Territories-Gaza/ West Bank	Palestinian households in the West Bank and Gaza Strip	Cross-sectional nationally representative household survey	Diabetes, hypertension, cardiovascular disease (CVD) and cancer	2013	4,456 households in the West Bank and 2118 in the Gaza Strip. The response rates for the 2 regions were 84.1% and 96.9% respectively	The authors emphasized that even though previous studies have combined Palestinians as one group, they live in different areas and are subject to different health systems which can result in different health outcomes. Being a refugee was a significant risk factor for diabetes and CVD while being married/engaged or divorced/ separated widowed was a risk factor for diabetes and hypertension. Non-refugees were 33% less likely to have diabetes and 46% less likely to have CVD than refugees
Abul (2001) [66]	Kuwait	Patients admitted to hospitals in Kuwait with asthma for six years (1987–1989 and 1992–1994)	Retrospective cross-sectional study	Asthma	2001	12,113 asthma patients during the pre-Gulf War period compared with 9,771 patients during the post-Gulf War period	During the war, a lot of oil wells were burned, giving suspicion to the potential for increase in asthma. No statistically significant difference in hospital admissions for to death rates attributable to asthma in the pre- and post-Gulf War periods in Kuwait. Notably, the war was 1990/1991, and no data is available for those years, so the immediate effect isn’t known
Ahmad (2015) [33]	Syria	Syrian national health system	Situational analysis using document analysis, key informant interviews, and direct clinic observation	Diabetes and cardiovascular disease (CVD	October 2009 -August 2010	53 semi-structured interviews	The rebuilding of a post-conflict heath care system in Syria may benefit from insights into the structural problems of the pre-crisis system. Weaknesses that existed before the crisis are compounded by the current conflict. The authors suggest an over reliance on secondary and tertiary care for DM patients with withdrawal of the Syrian government from the public health clinics, which led to escalating healthcare costs and fostered increasingly unequal access
Alabed (2014) [79]	Country of Asylum: Syria Country of Origin: Palestinian Territories	Palestinian refugees living in Damascus attending three UNRWA health clinics	Cross sectional	Diabetes	August 2008—September 2008	154 DM patients	UNRWA clinic inspections highlighted shortages in drug stocks with 47.3% of patients reporting problems accessing prescribed medications and 67.7% reporting having to buy medications at their own expense at least once since their diagnosis. Patients’ knowledge of their condition was limited, Patients were generally unaware of the importance of good glucose control and disease management. Women were more likely to attend the clinic than men, with 71% of patients being female
Ali-Shtayeh (2012) [80]	Palestinian Territories-West Bank	Patients attending outpatient departments at Governmental Hospitals in 7 towns in the Palestinian territories (Jenin, Nablus, Tulkarm, Qalqilia, Tubas, Ramalla, and Hebron)	Cross-sectional survey	Diabetes	August 2010—May 2011	1,883 DM patients	The use of CAM differed significantly between residents of refugee camps versus residents of urban or rural areas (*p* = 0.034). Those who were on CAM reported they were using it to slow down the progression of the disease or relieve symptoms. All patients with DM who used CAM were also on conventional therapies
AlKasseh (2014)[81]	Palestinian Territories-Gaza	Patients at UNRWA clinics within Gaza	Retrospective case–control study	Gestational diabetes (GDM)	March 2011—June 2011	189 postnatal GDM women with 189 matched controls by age and place of residency	The present study showed that history of miscarriage more than once, being overweight before pregnancy, history of stillbirth, history of caesarean birth and positive family history of diabetes mellitus were strongly correlated with developing GDM. The WHO criteria for screening for GDM remains a good instrument to identify GDM in refugee populations in war-torn countries (like the Gaza Strip)
Amini (2010) [98]	Iran	Completely blind Iranian survivors of the Iran-Iraq War	Cross-sectional study	Multiple NCDs including hypertension, Hypercholesterolemia, and erectile dysfunction	2010	250 Iran-Iraq war survivors	As blind war survivors’ age, they will present with a greater set of burdens despite their relatively better quality of life (QOL) in the physical component scale when compared with lower limb amputees. Risk factors of cardiovascular attack such as high blood pressure and hypercholesterolemia were present: High systolic and diastolic blood pressure, hearing loss, and tinnitus had negative individual correlations to (QOL) (*p* = 0.016, 0.016, 0.005, *p* < 0.0001). Hypercholesterolemia showed significant correlation to QOL (*p* = 0.021)
Bijani (2002) [105]	Iran	Iranians injured by chemical weapons during the Iraq–Iran war who are under services of the Mostazafan and Janbazan Foundations of Babol, Iran	Cross-sectional	Chronic respiratory diseases	1994—1998	220 patients	The clinical evaluations, radiography, and PFTs revealed that the most prevalent effects of chemical weapons on respiratory tract were chronic obstructive lung disease. Victims of suphorous gas had demonstrated involvement of airways during acute and chronic phases of injury, however over time clinical manifestations, radiography, and PFT gradually became normal. Most patients reported mustard gas exposure.. Chest X-Ray was not reliable to diagnose lung injury in these patients. Diagnosis was completed most accurately by PFTs
Ben Romdhane (2015) [85]	Tunisia	Tunisian national health system	Situational analysis	Cardiovascular disease and diabetes	2010	12 key informants were interviewed and eight documents were reviewed	Weaknesses that existed before the 2011 Revolution(Arab Spring) were compounded during the revolution. This study was conducted prior to political conflict but written post-conflict. Growth of the private sector fostered unequal access by socioeconomic status and reduced coordination and preparedness of the health system
Chan (2009) [106]	Pakistan	Patients ≥ 45 years who attended two different types of post-earthquake relief clinics during a 17-day field health needs assessment in response to the 2005 Kashmir earthquake	Comparative descriptive study	Multiple NCDs	February 2006 4 months post-earthquake	30,000 patients in a rural site, and 382 IDPs in a urban site	The greatest gap in health services post-earthquake in both sites was non-communicable disease management. Clinical records reviewed in all study locations showed a systematic absence of documentation of common NCDs. In rural areas, older women were less likely to receive medical services while older men were less likely to access psychological services in both sites. During days when solely male doctors provided clinical services in the rural site, medical services utilization decreased by 30%
Chan (2010) [99]	Pakistan	Face-to-face, household-based survey conducted 4 months after the 2005 Kashmir, Pakistan earthquake in internally displaced camps near Muzafarabad city	Cross sectional	Multiple NCDs	February 2006 4 months post-earthquake	167 households	Although the proportion of the population with chronic conditions was similar across these studied camps, 85% of residents in the smallest unofficial camp had no available drugs to manage their chronic medical conditions as compared with their counterparts residing in larger rural unofficial (40%) and official camps (25%)
Doocy (2013) [107]	Country of Asylum: Jordan/ Syria Country of Origin: Iraq	Iraqi populations displaced in Jordan and Syria	Cross-sectional	Disability and multiple NCDs including hypertension, arthritis, diabetes, chronic respiratory diseases, and cardiovascular disease	October 2008-March 2009	1200 and 813 Iraqi households in Jordan and Syria, respectively	Chronic disease prevalence among adults was 51.5% in Syria and 41.0% in Jordan, with hypertension and musculoskeletal problems most common. Overall disability rates were 7.1% in Syria and 3.4% in Jordan, with the majority of disability attributed to conflict and depression the leading cause of mental health disability
Doocy (2015) [108]	Country of Asylum: Jordan Country of Origin: Syria	Syrian refugees in non-camp settings in Jordan	Cross-sectional survey	Multiple NCDs including hypertension, arthritis, diabetes, chronic respiratory diseases, and cardiovascular disease	1994—1998	1,550 refugees	More than half of Syrian refugee households in Jordan reported a member with an NCD. Among adults, hypertension prevalence was the highest (9.7%, CI: 8.8–10.6). While care-seeking was high (85%) among those reporting a NCD, among those who did not seek care, cost was the primary reason
Ebrahimi (2014) [68]	Iran	Patients with cardiovascular and respiratory diseases who received medical services from the Center for Disaster and Emergency Medicine in Sanandaj, Iran during dust event days	Ecological study	Cardiovascular and respiratory diseases	March 2009—June 2010	–	The authors demonstrated a statistically significant increase in emergency admissions for cardiovascular diseases during dust storm episodes in Sanandaj, Iran(r 0.48, *p* < 0.05). The correlation between respiratory diseases and dust storm events were statistically insignificant (0.19)
Eljedi (2006) [88]	Palestinian Territories-Gaza	Diabetic patients who were recruited from three refugee camps in the Gaza strip with age- and sex-matched controls living in the same camps	Cross sectional	Diabetes	November 2003—December 2004	197 patients	Using the World Health Organization Quality of Life questionnaire (WHOQOL-BREF) four domains were strongly reduced in diabetic patients as compared to controls, with stronger effects in physical health (36.7 vs. 75.9 points of the 0–100 score) and psychological domains (34.8 vs. 70.0) and weaker effects in social relationships (52.4 vs. 71.4) and environment domains (23.4 vs. 36.2). The impact of diabetes on health-related quality of life was especially severe among females and older subjects
El-Sharif (2002) [69]	Palestinian Territories-West Bank	Schoolchildren aged 6–12 years attending 12 schools in the Ramallah District of the Palestinian West Bank	Cross-sectional	Asthma	Autumn of 2000	3,382 children	Children from refugee camps were at a higher risk of asthma and asthma symptoms than children from neighboring villages or cities. Physician-diagnosed asthma was almost double in refugee camps than other places (15.6% versus 8.1% in villages and 7.3% in cities, pv0.001)
Forouzan (2014) [70]	Iran	Patients presenting with asthma or bronchospasm in western Iran	Prospective observational	Asthma	November 2013	2,000 patients	Many patients presented with bronchospasm after a thunderstorm
Kallab (2015) [44]	Country of Asylum: Lebanon Country of Origin: Syria	Syrian refugees and vulnerable Lebanese being treated in 8 health facilities run by Amel Association International	Program implementation reflection	Diabetes and hypertension	November 2014- May 2015	1,825 patients	Of the 1,825 patients enrolled in the program hypertension and diabetes accounted for 46% and 27% of cases respectively, with the remaining 27% of patients presenting with both diseases. The program addressed two main problems in Lebanon: lack of access to NCD services and lack of proper management of NCDs. Major challenges included insecurity in the country, patient transportation cost, and high workload for providers
Karrouri (2014) [91]	Country of Asylum: Tunisia Country of Origin: Libya	Case of a 10-year-old Libyan boy	Case report	Diabetes	–	1 patient	Report of a 10 year old without personal or familial diabetes mellitus history who developed type 1 diabetes appeared immediately following severe psychological trauma
Khader (2012) [109]	Country of Asylum: Jordan Country of Origin: Palestinian Territories	Persons with DM at Nuzha PHC Clinic	Retrospective descriptive study of the cohort reporting framework to monitor burden of disease and management	Diabetes	October 2009- March 2012	2851 patients	A directly observed therapy (DOTS) cohort monitoring system can be successfully adapted and used to monitor and report on Palestinian refugees with DM in Jordan. A sizeable proportion of DM patients of the clinic failed to have postprandial blood glucose measurements, and BP measurements in those with comorbid HTN. The study demonstrated to the clinic that they were either not performing or not recording disease-specific procedures that should be done at the investigated visits—can now improve on these in the future and monitor thanks to e-Health system
Khader (2013) [110]	Country of Asylum: Jordan Country of Origin: Palestinian Territories	Palestine refugees living in Jordan	Descriptive cohort study using routine data collected through e-Health	Diabetes	October 2009- June 2013	12,549 total patients	High burden of disease with predicted annual additional caseload is over 1,000 patients with DM. Many indicated risk factors: smoking, physically inactive, and obesity. Those who came had relatively good disease control. Points to the importance of using e-Health systems to monitor and evaluate and use for strategic planning. Complications, including myocardial infarction and end-stage renal disease were significantly more common in males. Females were more likely to be obese
Khader (2014) [111]	Country of Asylum: Jordan Country of Origin: Palestinian Territories	Palestine refugees living in Jordan	Retrospective cohort study with program and outcome data collected and analyzed using E-Health	Hypertension	October 2009- June 2013	18,881 patients	Endorses the use of E‐Health and cohort analysis for monitoring and managing patients with HTN and DM. High case load from HTN and comorbid HTN and DM(40–50%) amongst Palestinian refugees being treated at UNRWA primary health care clinics in Jordan. Most common risk factors included smoking, physical inactivity, and obesity. 33% of males smoked, while more than 50% of the women were physically inactive. 75% of women were obese
Khader (2014) [104]	Country of Asylum: Jordan Country of Origin: Palestinian Territories	Palestinian refugees living in Jordan with DM attending Nunzha Clinic	Retrospective cohort	Diabetes	2010–2013	119 DM patients	The E-health system was successful in monitoring annual outcomes, measures of disease control, and development of complications in a cohort of patients with DM. Three major findings were: a progressive loss of patients attending the clinic, mainly lost to follow-up; routine measurements were always performed, and there was a progressive increase in late-stage complications, predominately due to cardiovascular disease and stroke
Khader (2014) [45]	Country of Asylum: Jordan Country of Origin: Palestinian Territories	Palestinian refugees living in Jordan with DM attending Nunzha Clinic	Retrospective cohort study	Diabetes	2012	2,974 DM patients	E-Health systems are useful for monitoring patients, since over half of patients who fail to attend a scheduled quarterly appointment are declared lost to follow-up 1 year later. This suggests a need for monitoring and active follow-up
Khan (1997) [59]	Country of Asylum: Pakistan Country of Origin: Afghanistan	Patients from North West Pakistan and Afghan refugees attending the Institute of Radiotherapy and Nuclear Medicine, Peshwar	Cross-sectional	Cancer	1990—1994	13,359 patients	In male Afghan refugees, esophageal cancer represented 16.6% of the cases, compared to only 4.6% of the cases in Pakistani residents. Similar patterns in women (13.1% vs. 4.1%)
Khateri (2003) [100]	Iran	Individuals with confirmed exposure to mustard agent during the Iran–Iraq war of 1980–1988 and who were evaluated for exposure to mustard agent by medical authorities	Retrospective Cohort	Chronic pulmonary, ocular, and cutaneous lesions	1997–2000	34,000 cases	Among patients, there was a high degree of pulmonary disease: 42.5% of the exposed population exhibiting chronic lung lesions and associated symptoms. Ocular damage, which is observed to be present in 39.3% of mustard exposed Iranians, is another major consequence of exposure to these agents as a result of their ease of absorption through the unprotected eye
Lari (2014) [72]	Iran	Patients exposed to sulfur mustard gas	Cross sectional	Chronic obstructive pulmonary disease (COPD)	March 2010—April 2011	82 patients	The COPD Assessment Test (CAT) was found to be a valid tool for assessment of health-related quality of life in chemical warfare patients with COPD
Leeuw (2014) [101]	Country of Asylum: Jordan, Lebanon Country of Origin: Syria	Syrian refugee households in Jordan and Lebanon	Cross sectional	Multiple NCDs	2013	3,202 refugees	Impairments found in 22% of refugees and disproportionately affecting those over 60 years of age (70% with at least 1 impairment)
Mansour (2008) [93]	Iraq	Patients struggling with diabetic control	Cross sectional	Diabetes	January 2007- December 2007	3,522 patients	Patient opinion for not achieving good glycemic control included the following: 50.8% cases reported no drug supply or drug shortage, while 50.2% reported high drugs and/or laboratory expenses. 30.7% percent of patients said that they were unaware of diabetic complications and 20.9% think that diabetes is an untreatable disease. 30% think that non-control of their diabetes is due to migration after the war. No electricity or erratic electricity, self-monitoring of blood glucose is not available, or strips were not available or could not be used, and illiteracy as a cause was seen in 15%, 10.8% and 9.9% respectively
Mateen (2012) [48]	Country of Asylum: Jordan Country of Origin: Iraq	Iraqi refugees receiving UNHCR health assistance in Jordan	Cross sectional	Multiple NCDs including hypertension, visual disturbances, diabetes, and joint disorders	January 2010-December 2010	7,642 registered Iraqi refugees	Among adults 18 years or older, 22% had hypertension; 11% had type II diabetes mellitus; 4% had type I diabetes mellitus; 10% had visual disturbances; 10% had disorders of lipoprotein metabolism and other lipidemias; 9% had other joint disorders and 7% had chronic ischemic heart disease. Cancer care was required by 2% of refugees. For all refugees as a group, the largest number of visits were for essential hypertension (2067 visits); visual disturbances (1129); type II diabetes mellitus (1021); other joint disorders (969), and acute upper respiratory infections (952)
McKenzie (2015) [62]	Country of Asylum: Jordan Country of Origin: Iraq, Syria	Iraqi/Syrian refugees residing in Jordan	Retrospective cohort	Neuro-psychiatric disorders	2012–2013	223 refugees	Among neuropsychiatric applications, stroke was the most common diagnosis, accounting for 16%. Brain tumors accounted for 13% of neuropsychiatric applications and was the most expensive diagnosis overall and per applicant. The ECC denied six applications for reasons of eligibility, cost, and/or prognosis. Of the 20 approved applications, 15% (*n* = 3) were approved for less than the requested amount, receiving on average 39% of requested funds
Mirsadraee (2011) [73]	Iran	Patients whose parents were exposed to chemical warfare	Case control	Asthma	–	409 children	The prevalence of asthma was not significantly different in the offspring of chemical warfare victims
Mousa (2010) [50]	Country of Asylum: Jordan, Lebanon, Syria, West Bank/Gaza Country of Origin: Palestinian Territories	Refugees registered by the United Nations Relief and Works Agency for Palestine Refugees in the Near East (UNRWA)	Case series	Diabetes and hypertension	June 2007	7,762 refugees	A total of 9% of those screened were diagnosed with hypertension or diabetes. Being older than 40 years, obese or with a positive family history of diabetes or cardiovascular disease increased the risk of presenting with hypertension and/or hyperglycemia 3.5, 1.6 and 1.2 times respectively. Risk factors were very common (obesity and smoking)
Otoukesh (2012) [51]	Country of Asylum: Iran Country of Origin: Afghanistan	Afghan refugees in Iran	Retrospective cross sectional	Multiple NCDs including ophthalmic diseases, neoplasm, nephropathies, ischemic heart disease, and perinatal disorders	2005 -2010	23,152 refugees	The Afghan refugees who received referrals for care represented a higher number of women, age 15- 59 years old, for ophthalmic diseases, neoplasms, and nephropathies
Shamseddine (2004) [64]	Lebanon	Lebanese population following the 1975 -1990 Lebanese Civil War	Nationwide, Population-Based Prevalence Study	Cancer	1998	4,388 cases	Among males, the most frequently reported cancer was bladder (18.5%), followed by prostate (14.2%), and lung cancer (14.1%) Among females, breast cancer alone constituted around one third of the total cancer caseload in the country, followed by colon cancer (5.8%), and cancer of the corpus uteri (4.8%). One limitation of the study is that the last and only census undertaken in Lebanon was in 1932, and the population estimates and projections may have been subject to minor inaccuracies
Sibai (2001) [52]	Lebanon	Lebanese aged 50 years and over residing in Beirut, Lebanon in 1983	Retrospective cohort study	Multiple NCDs including cancer, cardiovascular disease, cancer, and nephropathies	1983–1993	1,567 cases	The most important causes were non-communicable diseases, mainly circulatory disease (60%); and cancer (15%). Among circulatory diseases, ischemic heart disease accounted for the majority of the mortality burden (68%) followed by cerebrovascular diseases (21%). In countries that lack reliable sources of mortality data, the utility of verbal autopsy can be viably extended to cohort studies for assessing causes of death
Sibai (2007) [112]	Lebanon	Lebanese aged 50 years and over residing in Beirut, Lebanon	Retrospective cohort study	Cardiovascular disease	1984–1994	1,567 cases	Most important causes of death were CVD and Cancer. High adjusted risk of CVD mortality associated with being single (never-married) versus married among men and women
Sofeh (2004) [113]	Country of Asylum: Peshawar, Pakistan Country of Origin: Afghanistan	Afghan refugees attending Red Cross dispensaries and hospitals in Peshawar Pakistan	Cross-sectional	Multiple NCDs including diabetes mellitus	–	456 patients	Out of 456 patients examined during the study, 255 patients suffered from DM, 80 with hepatitis, 69 with nephritis, and 52 with hyperlipidemia
Strong (2015) [53]	Country of Asylum: Lebanon Country of Origin: Syria	Syrian refugees over age 60 residing in Lebanon and registered with either Caritas Lebanon Migrant Center (CLMC) or the Palestinian Women’s Humanitarian Organization (PALWHO)	Cross-sectional	Multiple NCDs including hypertension, diabetes, heart disease, hyperlipidemia, arthritis, and ocular diseases	March 2011—March 2013	210 refugees	Older refugees reported a high burden of chronic illnesses and disabilities. Hypertension was most common (60%), followed by diabetes mellitus (47%), and heart disease (30%). The burden from these diseases was significantly higher in older Palestinians compared to older Syrians, even when controlling for the effects of sex and age. Financial difficulties were given as the primary reason for not seeking care by 79% of older refugees
Wright (2010) [78]	Kuwait	Kuwaiti nationals ages 50–69 exposed to the 1990 Iraqi invasion	Cross-sectional	Asthma and PTSD	December 2003—January 2005	5,028 subjects	War-related stressors were associated with elevated risk of incident asthma in elderly Kuwaiti civilians exposed to 1990 Iraqi invasion. Study suggested that those who reported highest stress exposure in the invasion were more than twice as likely to report asthma. Suggestive of correlation between war trauma and asthma
Yaghi (2012) [97]	Lebanon	Cases of amputations in Lebanon	Cross- sectional	Diabetes	January 2007—December 2007	661 amputations	Diabetes and vascular indications were not only more common than trauma-related amputation, but both were associated with more major surgery and longer hospital stay including conflict afflicted southern Lebanon where trauma, diabetes and vascular disease amputations all occurred at more than twice the national rate
Yusef (2000) [83]	Country of Asylum: Lebanon Country of Origin: Palestinian Territories	Diabetic and hypertensive patients attending UNRWA primary health care facilities in Lebanon	Cross-sectional	Diabetes and hypertension	1997	2,202 records	Presence of both diabetes and hypertension increased the risk for late-stage complications. Only 18.2% of diabetic patients and 17.7% of diabetic patients with hypertension were managed by lifestyle modification. About 50% of type 2 and 66% of type 1 patients who were on insulin were well controlled. Medication shortages may drive medication choices for hypertension
Zubaid (2006) [57]	Kuwait	Catchment area of Mubarak Al Kabeer Hospital	Ecological	Acute myocardial infarction (AMI)	March 2003	1 Missile Attack Period (MAP) and 4 control periods	The number of admissions for AMI was highest during MAP, 21 cases compared to 14–16 cases in the four control periods, with a trend towards increase during MAP (incidence rate ratio = 1.59; 95% CI 0.95 to 2.66, *p* < 0.07).The number of admissions for AMI during the first 5 days of MAP was significantly higher compared to the first 5 days of the four control periods (incidence rate ratio = 2.43; 95% CI 1.23 to 4.26,*p* < 0.01). This indicates missile attacks were associated with an increase in the incidence of AMI

**Table 9:** Characteristics of included publications by region: Western Pacific

	**Country/ Territory of Interest**	**Target Population**	**Type of Study**	**NCD Studied**	**Years of Observation**	**Number of study participants**	**Major Findings**
An (2014) [82]	China	Adults who had lived through the 1976 Tangshan earthquake (vs control Age 37–60	Cross-sectional	Diabetes	Sept 2013 – Dec 2013	1551 adults	Earthquake stress linked to higher incidence of DM Women more likely to have diabetes after experiencing earthquake stressors compared to men
Chan (2011) [79]	China	Evacuees from the 2008 Sichuan earthquake	Descriptive, cross sectional study	Multiple NCDs including diabetes, hypertension, heart failure	May 2008	132 adults	Chronic health needs constituted a significant proportion of emergency care during the acute phase of the earthquake. Disaster responders must consider NCDs as well as trauma
Chen (2009) [40]	China	Adults who were in the West China Hospital on the day of the 2008 Sichuan (Wenchuan) earthquake	Case series	Hypertension	May 2008	11 patients	Mean blood pressure and heart rate increased immediately after the earthquake, regardless of gender or pre-existing hypertension. BP gradually declined within 6 h after the earthquake and increased again during aftershocks
Hung (2013) [42]	China	Patients treated by Hong Kong Red Cross three weeks after the 2008 Sichuan earthquake	Cross sectional chart review	Multiple NCDs including hypertension, stroke	June 2008	2,034 patient encounters	There was a high prevalence of chronic disease after the earthquake, especially hypertension
Huynh (2004) [58]	Vietnam	Women hospitalized with invasive cervical squamous cell carcinoma (subjects) and other extrauterine cancers (controls)	Case control	Cancer	June 1996 – Sept 1996	145 women in S. Vietnam, 80 women in N Vietnam	The development of invasive cervical cancer was significantly associated with military service by husbands during the 2^nd^ Indochinese War and with parity status. Geographic and temporal variation in cervical cancer rates among Vietnamese women was associated with the movement of soldiers
Li (2010) [85]	China	Rural adults born between 1954 and 1964 in selected communities from the 2002 China National Nutrition and Health Survey	Cross-sectional	Diabetes	2002	7,874 adults	Fetal exposure to severe famine increases the risk of hyperglycemia in childhood. The association is exacerbated by a nutritionally rich environment later in life
Li (2011) [102]	China	Rural adults born between 1954 and 1964 in selected communities from the 2002 China National Nutrition and Health Survey	Cross-sectional	Diabetes	2002	7,874 adults	Fetal or infant exposure to famine increases the risk of metabolic syndrome in adulthood. The association is exacerbated by a western dietary pattern or being overweight in adulthood
Li (2012) [114]	China	People exposed to the 1959–1961 Chinese famine and those not exposed	Retrospective cohort	Stomach cancer	1970–2009	Population level data: (2.4million)	Prolonged malnutrition during early life may increase the risk of stomach cancer later in life
Marom (2014) [61]	Philippines	People who survived the 2013 earthquake and typhoon (Haiyan) in the city of Bogo (northern Cebu island)	Case series	Head and neck tumors	2013	1,844 adults treated at the field hospital – 5% (85) had H&N tumors	Surgical interventions in pts with H&N tumors in a relief mission can be performed for therapeutic, palliative and diagnostic purposes
Read (2015) [67]	Philippines	Patients treated by an australian govt deployed surgical team in a field hosp in Tacloban for 4 wks after Typhoon Haiyan	Cross sectional	Diabetes	Nov 2013	131 people	Sepsis from foot injuries in diabetic patients constituted an unexpected majority of the workload of a foreign collaborative surgical medical team in Tacloban in the aftermath of Typhoon Haiyan
Sun (2013) [54]	China	Survivors of Wenchuan earthquake staying in a temporary shelter for more than 1 year	Cross sectional	Hypertension	March–May 2009	3,230 adults	Age, family history of hypertension, sleep quality, waist-to-hip ratio, BMI and blood glucose levels are risk factors for earthquake-induced hypertension. Mental stress was not a risk factor
Wagner (2016) [96]	Cambodia	Community health care workers in Siam Reap province	Cross sectional	Type 2 Diabetes	.	185	Community health workers were able to effectively learn diabetes prevention curriculum suggesting that they would be effective at disseminating the information
Wu (2015) [80]	China	Senior over age 60 living in 8 villages in China during a 2011 flood	Cross sectional survey	Multiple NCDs including hypertension, diabetes	February 2012	1,183 elderly patients	There was a marked decline in health status of the elderly after a flood. There were greater detrimental impacts on women and single elderly

**Table 10:** Characteristics of included publications by region: Southeast Asia

	**Country/Territory of Interest**	**Target Population**	**Type of Study**	**NCD Studied**	**Years of Observation**	**Number of study participants**	**Major Findings**
Gilder (2014) [89]	Country of Asylum: Thailand Country of Origin: Myanmar	Women attending the antenatal care (ANC) clinic in Maela refugee camp on the Thai–Myanmar border	Cross sectional	Diabetes	July 2011—March 2012	228 women	The prevalence of GDM is lower in this population compared with other populations, but still complicates 10% of pregnancies. Despite the weight of evidence for the benefits of early diagnosis and treatment of GDM, the absence of a simple, inexpensive and applicable screening method remains a major barrier to GDM screening programs in refugee camps and other resource-poor settings
Guha-Sapir (2007) [76]	Indonesia	Patients attending an International Committee of the Red Cross(ICRC) field hospital in Aceh, Indonesia, established immediately after the tsunami in 2004	Cross-sectional	Multiple NCDs including hypertension, diabetes, chronic respiratory diseases, trauma or injury, and psychiatric illness	January 15, 2004—January 31 2004	1,188 study participants	Chronic diseases including HTN and DM represented 43.5% of consultations including acute presentations of chronic illnesses. These cases presented soon after the hospital opened and accounted for about half of the consultations. The largest diagnostic groups included: respiratory diseases (21.0%), other chronic diseases, such as diabetes and hypertension (17.3%), trauma or injury (9.8%) and psychiatric illness (9.7%). Females’ odds of acute disease were 34% lower than males (95% CI: 16–49%, *P* = 0.001) however were 65% more likely than males to be diagnosed with a psychiatric illness (95% CI: 11–145%, *P* = 0.013)
Khan (2013) [115]	Bangladesh	Respondents living in flood and stagnant water-affected areas of Dhaka over 10 years of age	Cross-sectional	Multiple NCDs	March 2008 -March 2009	3,207 study subjects	Respondents living in flood and stagnant water-affected(FSW) areas were more vulnerable than their non-affected counterparts. While respondents living in the FSW-affected areas reported more communicable and poor mental well-being, the prevalence of NCDs was remarkably lower in the affected (2.7%) than the non-affected areas (4.8%). However, FSW affected area respondents also reported a lack of availability to be evaluated by a medical doctor, which may affect these results
Kunii (2002) [71]	Indonesia	Patients exposed to air pollution in the “haze disaster” in Indonesia following large forest fires throughout Indonesia	Cross sectional	Chronic Respiratory Diseases including asthma	September 1997 -October 1997	543 study subjects	Patients had increased respiratory issues after a large forest fire disaster, especially the elderly. Wearing a high-quality face mask was protective (vs handkerchief or simple surgical mask). Almost all of the respondents (98.7%) developed or suffered from an exacerbation of symptoms, and 91.3% had respiratory symptoms. Most of the health problems were mild, but 13.1% perceived their health problems as severe and 49.2% reported that the health problems disturbed their daily life
Molla (2014) [74]	Bangladesh	Children under 5 years of age categorized as climate refugees in Dhaka with diarrhea and asthma	Cross sectional	Asthma and diarrheal diseases	September 2012 -November 2012	410 households	Asthma caused a significantly higher number of disability adjusted life years (DALYs) lost in the group displaced due to climate change in comparison to controls. Associated contributing factors included overcrowding and improper household ventilation for domestic cooking or burning among climate change refugees
Ramachandran (2006) [94]	India	Tsunami affected population of Chennai(Madras) in Southern India aged 20 and above	Retrospective cohort	Diabetes	April 2005- June 2005	1,184 tsunami affected subjects, 1,176 controls	Undetected diabetes and impaired glucose tolerance were higher in the tsunami-hit area as compared to controls. Diabetes prevalence was found to be similar in the tsunami affected population and control group. Women reported higher stress scores and demonstrated a higher prevalence of impaired glucose tolerance as compared to their male counterparts
Redwood-Campbell (2006) [77]	Indonesia	Patients registering in the ICRC field hospital in Banda Aceh after the tsunami	Cross Sectional	Multiple NCDs including respiratory, psychiatric, endocrine, urological, and neurologic diseases	March 2005	271 patients	12% of the problems seen in the clinic 9 weeks after the tsunami were directly related to the tsunami. Majority of patients were male, the problems were urologic, digestive, respiratory and musculoskeletal in that order. 24% had 4 or more depression/ PTSD symptoms

### Cardiovascular disease

Regarding overall number of publications, cardiovascular disease was the most commonly studied NCD after diabetes, and 29 studies addressed this (Table 1, Fig. 1). Syrian refugees were the most commonly studied population among studies addressing CVD (Table 11) [44, 52, 53, 83].

**Table 11:** CVD Key Findings

Second most commonly studied NCD among results
Studies predominantly on CVD risk factors over disease outcomes
Studies demonstrate CVD is among leading diagnoses in these settings, including ischemic heart disease and stroke
Hypertension a leading risk factor, and disease control is poor
Gender differences in CVD outcomes exist in these settings, including divergence from conventional disproportionate burden for CVD among men
Exposure to disaster is associated with worse CVD outcomes
Refugee status confers worse outcomes
Scant study focus on management of disease

Prevalence of disease was high [32, 40, 52], as demonstrated by Sibai et al. in a community-based cross-sectional study of residents of Beirut, Lebanon with circulatory diseases accounting for nearly 60% of diagnoses, and ischemic heart disease was the leading diagnosis [52]. They also demonstrated that strokes had the second highest case fatality rate (54%), which was second only to sepsis (60%). However, most studies assessed cardiovascular disease risk factors, or intermediate risk factors [68], as opposed to actual diseases such as heart attack or stroke. Among cardiovascular disease studies, only two studies primarily addressed strokes [43, 46].

As highlighted, only two studies primarily addressed strokes [43, 46]. In one of the studies, which was conducted two years after the 1991–97 Croatia War in the Baranya region of Croatia, they found 513 stroke cases in a single-site emergency department study [46]. The patients had an average age of 68.4y, with an age range from 25-91y, and a near equal distribution of the cases between men and women (51.7% male). Only 50.6% of patients presented within 6 h, another 16.2% presented after 24 h [46], paresis, speech impairment and vision impairment were the most common presenting symptoms. 38.4% died in hospital. 85.8% of patients had hypertension, 27% had diabetes, 44.6% had hyperlipidemia and 46% also had cardiac disease.

As far as risk factors, hypertension was the lead CVD risk factor in several studies [42–44, 47, 50, 53, 54, 112], and reason for presentation for care among refugees. This was evident for Iraqi refugees in Jordan where, for adults 18 and older, primary hypertension was the top diagnosis [48]. However, blood pressure control remains a problem, as demonstrated among victims staying in temporary shelter more than 1 year after a 2008 earthquake in the Sichuan province of China, where only half of those diagnosed had medications (53.4%) [54] and less than one in five (17.8%) demonstrated control.

Regarding CVD risk factors along gender lines, generally men tend to have a higher prevalence of hypertension as compared to women, and associated CVD (myocardial infarction, congestive heart failure, and stroke) [45, 51, 52]. However, in several studies we found a trend of disproportionate prevalence and worse outcomes for women for a variety of CVD outcomes [42, 45, 50, 83, 87]. Those citations with observed gender differences are described in detail in a separate review [116].

In turn, the effect of being exposed to a disaster was demonstrated to be a primary contributor to developing CVD in several studies [32, 34, 37, 39, 49, 55, 56]. In a Croatian study assessing the patterns of presentations for acute myocardial infarction (AMI) in 3,454 patients, they found a 23% increase in presentations during the war (1,254 vs 1,024 hospitalized patients) as compared to the 3-year period preceding the war of 1989–1991, and a 15% increase (1,173 hospitalized patients) as compared to the 3-year period even after the war (1995–1998) [49]. The incidence of hypertension and heart disease was also affected by those with death or injury in their family from disaster, with disease occurring most commonly within the first six months after an event [34]. In another study on residents affected by the Bosnian war, they assessed incidence of AMI and unstable angina (UA) 5 years prior to, during, and 5 years after the war [36]. The overall incidence of both AMI (*n* = 428 vs 365, *p* = 0.025) and UA (*n* = 185 vs 125, *p* = 0.001) was found to be higher during the war as compared to the period prior. In a Kuwait missile attack, Zubaid et al. found that the incidence rate of AMI hospital presentations more than doubled (incidence rate ratio = 2.43; 95% CI: 1.23 – 4.26, *p* < 0.01) for one year after the event [57]. Another study assessing the effects of dust storms in western Iran, showed there was an increase in cardiovascular events with a 1.35% increase in incidence of events for every 100 μg/m3 increase in the PM10 concentration (particulate matter greater than 10 μm) (*p* < 0.05) [39]. Finally, a retrospective cohort study assessing the effects of famine during the Biafran war (1967–1970) demonstrated association between undernutrition and the presence of hypertension, glucose intolerance, and overweight in Nigerian adults affected [41].

Furthermore, refugee status was associated with higher prevalence of CVD as compared to non-refugee counterparts in several studies. Abukhdeir et al. demonstrated a lower prevalence of CVD among those reporting non-refugee status in a representative sample of Palestinian households within the West Bank and the Gaza Strip (OR 0.539, *p* < 0.001), as compared to their refugee counterparts [32]. Yusef et al. highlighted an alarming predominance of late presentations for CVD, and other NCD risk factors, at United Nations Relief and Works Agency (UNRWA) primary health care facilities in Lebanon with 42% of respondents having at least one complication (such as retinopathy, nephropathy, and neuropathy) [83]. Similarly, Kadojic demonstrated that displaced persons in Croatia residing in camps had higher prevalence of hypertension, hyperlipidemia and obesity when compared to age-matched controls in settlements not impacted by the war [43].

Only one study assessed management of disease. This was a descriptive analysis by Yusef et al., showing that among refugees accessing care at UNRWA facilities in Lebanon, only 3% were on first-line anti-hypertensive therapy, up to 14.2% were on third line treatment, and 10% reported lifestyle modifications [83]. Another study discussed a complex intervention that included capacity-building of staff, provision of key diagnostic tools such as blood pressure cuffs, stethoscopes and glucometers), and advocacy on providing NCD care. The intervention took place in Lebanon [44], and they implemented screening for DM and hypertension in those 40y and older attending any of the clinics (five health centers and three mobile units), with the potential for referral to a specialist, such as cardiology, in case of need. This and the scant other interventions found in our study [38, 44, 61, 62, 67, 86, 96] are further described in a separate publication [31].

### Cancer

Multiple studies demonstrated that cancer and oncological emergencies affect populations in conflict (see Table 2). Of the ten articles included, there was a predominant geographic focus on the EMRO region. In Lebanon, Sibai et al. [52] observed that cancer was second only to cardiac disease as a cause of death. Cancer represented 15% of all causes of deaths in their retrospective cohort study of 1,567 Lebanese aged 50 years and over residing in Beirut during the Lebanese Civil War (1975–1990). This was followed post-war by Shamseddine et al. [64] who identified an overall crude incidence rate for all cancers combined of 141.4 per 100,000 among males and 126.8 among females, a sharp contrast to earlier estimates made in 1966, of 102.8 and 104.1, respectively [64]. Of note, few studies addressed refugees, Internally Displaced Persons (IDPs) or noncombatants, in particular [52, 64]. We identified no articles relating to cancer prevalence among refugees in Africa, Asia, or the Americas. No studies addressed palliative care for oncology patients in the disaster setting (Table 12).

**Table 12:** Cancer Key Findings

EMRO region with predominant focus among published studies
No studies addressed palliative care
Cancer predominant cause of mortality, second only to CVD in cases, and prevalence is increasing
Breast cancer, lung cancer, bladder cancer leading contributors with tobacco use as key underlying target; brain cancer and head/ neck cancer also cited
Gender disparity in cervical cancer incidence observed; associated with migratory nature of partners during conflict
Scant studies on cancer focused on refugees or internally displaced persons, rather focused on host population
Divergence in epidemiology of cancer among refugees as compared to host population where assessed
Refugee status and cost of care confer additional challenges for cancer care in these settings

Multiple studies indicate a high prevalence of modifiable cancer risk factors [60, 63–65] in conflict-affected populations that could be targets for future intervention such as Human papillomavirus (HPV) vaccination, anti-tobacco smoking campaigns, and access to adequate nutrient-rich food. Cervical cancer, in particular, was identified as being related to or affected by war [58, 63]. For example, in the study by Huynh et al. [58], they demonstrate that southern Vietnamese women whose husbands served in the armed forces experienced a more than 160%-290% increase in cervical cancer risk, relative to women whose husbands had not served in the armed forces. The authors attribute the association between male combat activity and cervical cancer as men become reservoirs of high risk subtypes of HPV which cause cervical cancer, acquired during wartime movement patterns [58, 117].

We also found a variety of tobacco-related cancers. Shamseddine et al. [64] found in reviewing 4,388 new cancer cases in post-civil war Lebanon, that lung cancer was the third most prevalent cancer type. In addition, they highlight that bladder cancer incidence rates are disproportionately higher in Lebanon than in the region, and globally. Breast cancer was listed by multiple studies as the most significant cancer burden amongst women in conflict affected LMICs—including studies relating to Lebanon [64], Afghanistan [59], and Pakistan [59]. Tobacco associated cancers were noted as prominent in multiple conflict affected nations and as amenable to prevention efforts through anti-smoking campaigns [59, 64].

Malnutrition in early life had demonstrated association with stomach cancer mortality for survivors of the 1959–1961 Chinese famine [60]. Birth cohorts of Zhaoyuan County, China who were exposed to famine or experienced malnutrition had stomach cancer mortality rates around twice as high as birth cohorts not exposed to malnutrition 15 to 20 years post-famine [60]. Proposed mechanisms by the authors for this relationship include a correlation between nutritional deficiency and H. Pylori infection, consumption of foods associated with development of gastric carcinoma in times of famine such as salted meat containing N-nitrosamines or nitrite, vitamin deficiencies, and heavy alcohol use [60].

Relating specifically to refugees, Otoukesh et al. [51], provided cancer prevalence data for refugees in a 2012 retrospective cross-sectional study of Afghani refugees residing in Iran. Using demographic and medical data collected between 2005 and 2010 from referrals to the United Nations High Commissioner for Refugees (UNHCR) offices in Iran for Afghani refugees, they found that neoplasms represented 13.3% of all referrals second only to ophthalmic diseases. Likewise, McKenzie et al. [62] found that amongst UNHCR registered Iraqi and Syrian refugees in Jordan, brain tumors accounted for 13% of all neuropsychiatric applications. Furthermore, Khan et al. found a divergence in the epidemiology of cancer diagnosis from the host population when compared to refugees, with esophageal cancer representing 16.6% of oncological cases amongst male Afghan refugees compared to only 4.6% of cases amongst Pakistani residents [59], and further evidence shows a difference in breakdown by ethnicity exemplified by Pashtun refugees who experienced a disproportionate frequency of referrals for oncologic disease (17%) amongst Afghani refugees residing in Iran despite receiving only two percent of all referrals [51].

Further studies identified challenges specific to refugee populations or subgroups of refugee populations [51, 58, 59, 61, 63]. Marom et al. [61] described clinical and ethical dilemmas in patients with head and neck cancers presenting to a joint Israeli-Filipino field hospital during the subacute period following a 2013 typhoon in the Philippines. They highlight the importance of awareness of cancer epidemiology in the target country prior to deployment. In this case, it guided the Israeli team’s clinical management such as prioritizing physical examination for cervical nodal metastases based on known prevalence of regional lymph node involvement at presentation in 70% of Filipinos with head and neck cancers [61].

Cost of care as a barrier for refugees with cancers was studied by McKenzie et al. [62] who aimed to assess the prevalence and cost of neuropsychiatric disorders among Syrian and Iraqi refugees requiring advanced specialty care in Jordan. The UNHCR funds tertiary level medical care for refugees based on the cost and acuity of required care by means of application to an Exceptional Care Committee (ECC). In reviewing refugee applications for tertiary care to the ECC, McKenzie et al. [62] found that brain tumors represented the most expensive neuropsychiatric diagnosis overall ($181,815 USD, $7,905 USD/ applicant). Other referral diagnoses were stroke, psychiatric diagnoses, trauma, infectious diseases, multiple sclerosis, neurodevelopmental abnormalities, and epilepsy.

### Chronic respiratory disease

Of the fourteen articles that addressed chronic respiratory disease, six were related to war, and most addressed health hazards faced by refugees or victims of chemical weaponry (see Table 3). The geographic focus of most of these studies was the Middle East, with six studies from Iran alone (Table 13).

**Table 13:** Chronic respiratory disease Key Findings

EMRO region with predominant focus among published studies
Chemical weaponry predominant contributor to chronic respiratory disease incidence and exacerbations of chronic disease in these settings
Natural disasters, specifically storms, fires and volcanic eruptions, also confer increased incidence of respiratory disease
Refugee status confers worse chronic respiratory disease outcomes

Two studies conducted in Kuwaiti patients affected by the Gulf War demonstrated the association between war trauma and increased incidence of asthma exacerbations. However, despite the increase in frequency, there was no change in severity of exacerbations [66, 78]. One study found increasing levels of self-reported stress exposure were correlated with reports of asthma [78]. In contrast, a chart review on patients admitted with asthma in Kuwait found no difference in admission or mortality rates from asthma when comparing the pre-war and post-war periods [66].

Chemical agents used during warfare, such as sulfur mustard gas, confer an additional risk for chronic respiratory disease [105]. In one study assessing incidence of asthma among children of individuals exposed to chemical warfare, a similar incidence of disease was found to that of individuals born to parents with asthma [73]. The comparable incidence is concerning for chemical warfare as an independent contributor to the development of asthma. Additionally, a cross-sectional study of a Chronic Obstructive Pulmonary Disease (COPD) cohort demonstrated increased morbidity of patients exposed to sulfur mustard gas also conducted in Iran, and validated use of the COPD Assessment Tool (CAT) for quality of life in this population [72].

The effect of storms on respiratory illness was also studied [39, 70]. The only prospective observational study within our review on chronic respiratory disease was on this topic, evaluating asthma exacerbations and bronchospasm associated with thunderstorms in southwestern part of Iran, Ahvaz [70]. Two thousand patients who presented with these complaints within three weeks of a thunderstorm were surveyed. This represented an abnormal surge in such complaints for emergency departments there. 30% of patients reported developing their symptoms on the day of the thunderstorm, although only 2% presented within 24 h. At 3 weeks follow-up, more than two thirds were still using medications, with beta-agonists being the most likely prescriptions, and corticosteroids following. More than half (51.7%) had no prior history of respiratory disease or complaints of shortness of breath. A retrospective chart review similarly looked at respiratory illness and evaluated correlation with dust storms [39]. In contrast, this study concluded that cardiac (*P* < 0.05), but not respiratory, disease was associated with occurrence of dust storms.

Beyond storms, a variety of studies looked at the health effects of different types of natural disasters via chart review of patients who presented after the disaster. A large forest fire in Indonesia caused a “haze disaster” in 1997 resulting in increased respiratory complaints [71]. Among 543 respondents, while only 7.4% had a history of chronic respiratory illness (asthma), 98.7% presented with respiratory complaints [71]. 49.2% of all respondents reported symptoms which disturbed their daily life [71]. In Ecuador, researchers looked at pediatric emergency department visits and found that there was an increase in frequency of visits associated with volcanic eruptions. Visits for asthma and asthma-related conditions doubled (RR 1.97, 95% CI 1.19, 3.24) during the three weeks following volcanic activity [75]. Among NCD presentations to an International Committee of the Red Cross (ICRC) Hospital in Banda Aceh, Indonesia post-tsunami respiratory diseases were one of the most commonly recorded conditions (21%), which included acute asthma exacerbations [76]. Similarly, Redwood-Campbell et al. [77] cited respiratory complaints as constituting 12% of presentations in the outpatient/ emergency department at the same Indonesian ICRC facility, with asthma making up 29% of those cases.

Studies looking at populations in refugee camps were epidemiologic in nature. In the Palestinian West Bank, children from refugee camps were at higher risk of asthma than children from neighboring villages or cities [69]. Having a history of wheezing was reported for 22.1% of children in refugee camps versus 16.5% in cities, and 15.5% in villages. Overall, 8.8% (*n* = 298) of children reported wheezing in the previous year, with a 17.1% lifetime prevalence of wheezing [69]. Similarly, in the slums of Dhaka, Bangladesh, children under 5 who were part of a “climate refugee” community were studied and compared to a non-refugee group. Asthma caused a 1069-fold higher number of disability adjusted life years (DALYs) lost in the group displaced due to climate change in comparison to non-affected populations [74].

### Diabetes

We found that studies addressing diabetes were predominantly conducted in the EMRO Region (see Table 4). Specifically, 20 studies were conducted in the Eastern Mediterranean Region, two studies were conducted in the Caucasus region, three studies occurred in Sub-Saharan Africa, six studies occurred in Asia including South and Southeast Asia, and two studies were conducted in Eastern Europe (Table 14).

**Table 14:** Diabetes Key Findings

DM is most commonly studied NCD
EMRO region with predominant focus among published studies
Reported association between stress and increased incidence of diabetes as well as impaired fasting glucose
Food insecurity in these settings contributes to challenges with diabetes management given lack of availability of meals
Malnutrition also a determinant for diabetes among children exposed in utero
Additional challenges for diabetes care include lack of access to medications and diagnostics, limited access to clinical sites for care and lack of patient understanding on disease management
Disease complications are a common cause for presentation, including but not limited to, being the lead cause for limb amputation surpassing those due to trauma
Being female, unmarried, having less education, additional comorbidities, and having refugee status confers worse outcomes for DM
Reliance on established tertiary care for diabetes management contributes to worse outcomes during crisis given reduced access to care

Multiple studies point to the relationship between stress and personal loss incurred in natural disasters and conflict, and a subsequent rise in occurrences of impaired fasting glucose (IFG) and diabetes mellitus (DM) among survivors [34, 38, 82, 91, 94]. One such retrospective cohort study by An et al. [82] investigated the long-term impact of stresses from the 1976 Tangshan earthquake on the occurrences of impaired IFG and DM among survivors and found that the incidences of IFG and DM for the exposure groups were significantly higher than that for the control group (*P* = 0.043 for IFG; *P* = 0.042 for diabetes), with those who had lost relatives exhibiting a higher diabetes incidence than those who had not lost relatives. This effect was only statistically significant in women earthquake survivors (*p* = 0.009) [82]. In addition, refugees with diabetes were found to have strongly reduced quality of life (HRQOL) as compared to age-matched non-diabetic controls as identified by Eljedi et al. using the World Health Organization Quality of Life questionnaire (WHOQOL-BREF), with particularly severe effects noted among females (*p* < 0.05 in all four domains) [88].

Additionally, several studies addressed food insecurity, and identified it as a primary contributing factor affecting diabetes management [38, 50, 53, 95]. A study focusing on older Palestinian refugees [53] found that participants practiced reduced meal portion sizes, skipping a meal, or foregoing a full day’s meals due to food shortage at a significantly higher rate than an age matched host population in Syria (reducing portion sizes *p* < 0.001; skipping a meal *p* < 0.001; not eating at all *p* < 0.001). Factors associated with skipped meals or reduced portion sizes included low economic status, larger household size, and type of residence (financial status *p* = 0.009; household size *p* < 0.001; type of residence *p* < 0.001). The number of days older refugees reported eating only bread and nothing else corresponded to reported financial status (*p* = 0.036). The authors theorized that food insecurity may result in challenges in the management of diabetes [53].

Further studies specifically addressed effects of fetal exposure to malnutrition and impaired glucose tolerance or diabetes later in life [41, 85, 90, 92]. Hult et al. [41] examined the accumulated risk for glucose intolerance 40 years following fetal exposure to famine in Biafra, Nigeria during the Nigerian civil war. The crude odds ratios for both impaired glucose tolerance and diabetes diagnoses were significantly higher for the group exposed to fetal or infant famine in comparison to controls [41]. Consistent findings were identified by a retrospective cohort study from China by Li et al. [85], who also identified a relationship between the severity of famine for fetal exposed subjects and risk of hyperglycemia later in life (OR = 3.92; 95% CI: 1.64–9.39; *P* = 0.002). Similarly, in a region of Northern Ethiopia recently affected by severe famine, clinical features of 100 insulin-treated diabetic patients were consistent with previous descriptions of malnutrition-related diabetes mellitus (MRDM): young age of onset (70% < 30 yrs), low BMI (mean 15.8), and resistance to ketosis (only 4% admitted with diabetic ketoacidosis despite 48% reporting insulin treatment interruption) [90].

Additional barriers to glycemic control in patients affected by conflict were: migration after war, lack of self-monitoring glucose strips, lack of access and cost of medications, failure to adequately screen for diabetes, inability to travel to a heath facility, lack of education regarding diabetes complications and management, food availability, and difficulty following patients over time [38, 45, 50, 53, 79, 83, 84, 86, 89, 90, 93, 104, 109–111]. One cross sectional study [93] which aimed to identify barriers to glycemic control from the patient perspective in a diabetic clinic in the south of Iraq, found that lack of drug supply from a primary health care center or drug shortage is a barrier for 50.8% of patients, while drug and/or laboratory expenses were a barrier for 50.2% of patients. 30.7% of patients said that they were not aware of possible diabetic complications and 30% thought that their failure to control their diabetes was due to migration after the war. Lack of electricity, lack of access to blood glucose monitoring devices, and illiteracy as a cause were cited by 15%, 10.8% and 9.9% respectively [93]. In Mali and Ethiopia, insulin was not widely available and access was limited by cost (US$ 11 per vial in Mali) [86, 90]. Multiple studies noted that syringes and self-monitoring blood glucose devices were not readily available and posed a financial burden to those who required access to them [84, 86, 90].

Diabetic limb amputations were also found to be highly prevalent amongst populations in disaster affected LMICs [67, 83, 97, 110] corresponding to low rates of diabetic foot examinations in refugee settings (e.g., Palestinian refugee diabetic patients’ feet were examined in only 8% of encounters at a UNRWA clinic) [79, 109]. In Lebanon, during the 2006 Lebanese–Israeli conflict, diabetes was the main indication for limb amputation (59%), followed by vascular disease (18%), and trauma (12%), with the highest amputation rates reported in the region experiencing the greatest conflict burden (3.82 per 10, 000 persons) [97]. Diabetic patients were older (mean age 73 years versus 30 years), more likely to have major surgery (OR = 7.87; 95% CI: 2.83–21.9), and stay in hospital longer (RR = 4.56, 95% CI: 2.41–8.64) than patients with trauma-related amputations [97]. Other complications of late stage disease were also prevalent, as demonstrated by Khader et al. in a community-based cross-sectional study of Palestinian refugees in Jordan with 10–20% of diabetic patients presenting with late stage complications of diabetes including blindness, cardiovascular disease, and limb amputations [109].

One study investigated complementary and alternative medicine (CAM) use among Palestinian diabetic patients and found the use of CAM differed significantly between residents of refugee camps as compared to residents of urban or rural areas (*p* = 0.034) [80]. Those who were on CAM reported they were using it to slow down the progression of disease or relieve symptoms and 68% of patients interviewed reported not disclosing CAM use to their physician or pharmacist.

While no study specifically aimed to focus on gender in their primary research objectives, we found a relationship between gender and prevalence or access to resources for diabetes, emerged as a recurring theme [32, 82, 83, 88–90, 94, 113, 118]. These findings will be presented in a separate publication [116]. Other common risk factors associated with diabetes type 2 included age, having a higher BMI, being divorced/widowed/separated, having never attended school, illiteracy, comorbid hypertension, hyperlipidemia, family history, sedentary lifestyle, history of traumatic exposure, and refugee status [32, 50, 53, 81, 83, 91, 97, 113].

Several studies also took a health systems approach and found that reliance on tertiary care for diabetes management fostered unequal access by socioeconomic status, geographic location, and escalating healthcare costs overall [33, 35, 84, 86]. One study from Georgia [84], which sought to identify the extent to which the Georgian health system provides for effective diabetes control post-independence, identified a systems level concern that only tertiary-level endocrinologists were able to modify treatment regimens and prescribe insulin whereas even endocrinologists who worked in polyclinics were unable to determine insulin regimens or prescribe insulin. Three studies from Syria [33], Tunisia [35], and Mali [86] identified a similar shift of diabetes care to the tertiary level prior to the emergence of conflict in these countries due to an emerging private sector [33, 35] and lack of specialists [86], respectively. In Mali, the lack of specialists was augmented by a lack of available guidelines, treatment protocols, and training for primary care level providers which prevented a transition of care to primary or general practitioners [86]. The authors theorized that this shift of diabetes care to the tertiary level contributed to reduced care access during active conflict in these countries [33, 35, 86].

### Other NCDs

Studies investigating other NCDs centered on musculoskeletal and joint disorders [34, 42, 48, 77, 119] epilepsy and other neuropsychiatric disorders [51, 62, 103], ophthalmic diseases [48, 51, 100], nephropathies and urologic complaints [48, 51, 77] (see Table 5). Two studies measured mortality rates [34, 52] and two also studied quality of life [98, 101]. The effects of disability were briefly touched on by Leeuw et al. [101], with Amini et al. [98] further identifying hearing loss, and tinnitus as having negative impacts on quality of life among blind survivors from the Iranian War (*p* = 0.005, *p* < 0.0001) as compared to non-afflicted counterparts. We found that the majority of the studies on other NCDs did not refer to specific diseases or illnesses [42, 48, 77, 99], but rather represented epidemiological studies referring to conditions more broadly such as in the case of Mateen et al. [48] referring to “joint disorders”, and Hung and Redwood-Campbell describing “musculoskeletal”, “respiratory complaints,” and “gastrointestinal complaints” of unclear etiology [42, 77].

Hung et al. described musculoskeletal complaints constituting 30.4% of presentations among those visiting a Hong Kong Red Cross clinic in rural China following the 2008 Sichuan earthquake [42]. Mateen et al. conducted a far-reaching study of refugees in 127 camp settings across 19 countries and found that reportable neurologic diseases accounted for 59,598 visits over a 4-year period [103]. Nearly 90% of these cases were for epilepsy, which they highlight far outweighed the prevalence of neurological diagnoses of an infectious nature. Another study investigated neuropsychiatric disorders among Syrian and Iraqi refugees in Jordan via retrospective review of applications to the Jordanian Exceptional Care Committee, and found stroke to be the most common neuropsychiatric diagnosis (*n* = 41 applications, 16% of neuropsychiatric applications; median age 64 years) [62].

Specific ophthalmic diseases identified by Mateen et al. include cataracts (1.44 visits per refugee) and glaucoma (1.46 visits per refugee), which were exceeded only by cerebrovascular disease (1.46 visits per refugee) among Iraqi refugees in Jordan [48]. Of note, more than half of the refugees received concomitant diagnoses in one visit. Otoukesh describes ophthalmic disease as the most common health referral (13.65%) for those aged 15–59 among Afghan refugees in Iran [51]. Amini et al. [98] measured Quality of Life (QOL) scores in Iranian survivors totally blinded during the Iran-Iraq War, the effects of which were mitigated among those with higher levels of education (*p* = 0.006). Urologic complaints were identified as predominant in the ICRC hospital in Banda Aceh, Indonesia with 19% of complaints [77]; specific examples of urologic disorders from Mateen et al. among Iraqi refugees constituting a significant amount of morbidity were prostatic hypertrophy and nephrolithiasis [48]. Hematologic disorders were described by Otoukesh, and the type of disorder varied by ethnicity, with referrals for the Baluch being the highest at 25% [51].

### Concomitant affliction with NCDs

Finally, co-affliction with multiple NCDs was a recurrent issue in our findings. This was demonstrated by Strong et al. among Palestinian refugees in Lebanon, with an average of 4 NCDs per person; Syrian refugees in the same study had an average of 2.5 NCDs per person [53]. Three or more risk factors were also seen in displaced persons in Croatia, a statistically significant difference in prevalence when compared to age-matched controls who were not displaced [43]. Clustering of risk factors was also evident in a populations being served by UNRWA in Jordan, Syria, Lebanon, West Bank, and the Gaza Strip, and the risk of having CVD was 2.7 times higher in individuals with 4 risk factors as compared to those with only 1 risk factor [50]. Concomitant affliction also conferred worse outcomes among Palestine refugees in Jordan with CVD (myocardial infarction, congestive heart failure, stroke and blindness) among those with hypertension and diabetes, when compared to those with hypertension alone in the same cohort (*p* < 0.01) [45]. Yusef et al. also demonstrated that having concomitant risk factors (such as diabetes and hypertension) resulted in a higher likelihood of presentation with late complications of NCDs at a UNRWA primary healthcare field site in Lebanon [83].

## Discussion

NCDs represented a significant burden for populations affected by humanitarian crises and natural disasters for all regions [40, 51, 52, 57, 58, 61, 62, 64, 74, 86, 89, 97, 106, 120] and even conferred increased mortality and morbidity when compared to infectious diseases in one study [40]. Diabetes was the most commonly studied disease, even exceeding cardiovascular disease despite a higher global epidemiologic burden of the latter [29]. Late stage complications of cardiovascular diseases and diabetes including stroke [40, 46, 62, 103, 110], diabetic foot amputations [67, 83, 97, 110], and myocardial infarctions [55, 57] were described in all regions [83, 110]. However, studies addressing chronic respiratory diseases and cancer were noticeably lacking. All the same, studies on cancer highlighted tobacco as a key underlying factor, which contributes to predominant cancer etiologies in these populations such as breast, lung and bladder cancer [59, 64]. Musculoskeletal and joint disorders [34, 42, 48, 77, 119] epilepsy and other neuropsychiatric disorders [51, 62, 103], ophthalmic diseases [48, 51, 100], nephropathies and urologic complaints [48, 51, 77] constituted additional commonly encountered NCDs, and co-affliction with multiple NCDs or NCD risk factors should be expected in care [43, 45, 50, 53, 83]. As far as regional focus of studies assessed, both Sub-Saharan Africa and the Americas were poorly represented in the literature on NCDs in humanitarian crises [40, 41, 86, 90] (Tables 6 and 7). This is in spite of the fact that these regions experience a marked dual burden of armed conflict [115, 121] and natural disasters [122, 123], and represent a significant portion of the global NCD burden [107, 108]. Several studies demonstrated that NCDs adversely affected morbidity of populations in humanitarian crises with women and older populations disproportionately affected [72, 88, 98, 124]. Meanwhile, scant studies focused on pediatric populations, another vulnerable population [69, 73–75]. Strengthening and broadening the spectrum of NCD diagnoses included in disaster management planning is key, and particular focus on children and adolescents is critical as these age groups present key opportunities for interventions to mitigate future NCD morbidity [114, 125]. Several studies identified challenges and epidemiologic factors specific to refugee populations or subgroups of refugee populations [51, 58, 59, 61, 63], and they also highlight that refugee populations are heterogenous in their disease burden as compared to the host population. Understanding these sub-populations is key in guiding the medical equipment, personnel including specialists, and potential screening programs that should be considered in future humanitarian efforts. Assessing contributors to disease development and progression to complications, in prospective studies, would also be beneficial. Additionally, studies are needed on effective management of NCDs in these settings including for intervention implementation, which were notably lacking but critical in these uniquely resource-constrained contexts. Finally, policies that ensure established host country capacity for NCD care in addition to those for disaster response agencies that incorporate guidance for NCD care are key.

### Further research, policies and interventions needed for lead four NCDs among diverse populations in disaster settings

There was a predominant focus on diabetes, including among studies from the EMRO region which constituted the region with the highest number of publications. For studies on DM, 57.6% of studies were conducted in that region alone. Furthermore, 32.4% of DM studies focused on the Palestinian population alone [32, 50, 79–81, 83, 88, 104, 109–111], higher than Africa, the Americas, Western Pacific (WP), and South East Asia (SEA) combined. The high prevalence of articles conducted in the EMRO may reflect the higher prevalence of diabetes there [126]. However, with a rise of diabetes in all regions including Sub-Saharan Africa, where the largest percentage increase in the incidence of diabetes is projected in the coming decade, this represents a significant gap in the available literature [127]. Increased research on diabetes in these understudied regions is particularly needed on interventions targeting screening and early disease recognition in order to forego complications, highlighted in several articles [67, 83, 97, 109, 110]. Additionally, best practices on management of disease such as through controlled trial designs would be ideal. Innovative interventions are needed given limited access to self-monitoring devices, insulin as well as potentially limited health literacy [84, 86, 90, 93]. Implementation of clinical policies as well as education for providers and patients are important [128]. Additionally leveraging of community leaders [129], as well as more novel interventions such as the poly-pill [130] and mHealth [131] may be potential opportunities for treatment in these highly limited resource-variable settings.

Further studies are also needed on the additional leading NCDs, particularly cancer and chronic respiratory disease; this includes on epidemiology to guide policy and further research. For example, the number of studies on CVD were surpassed by those on DM, despite the significant global public health burden as the leading cause of death [29]. Understandably, CVD may be more challenging to diagnose, screen and test for, but that does not mean that efforts should not be made to do so in these contexts. Moreover, disease focus lacked as compared to the primary focus on risk factors [42–44, 47, 50, 53, 54, 112]. To that end, a focus on risk factors is laudable given potential for guided interventions that target prevention, however, understanding the epidemiology of disease is also important in order to effect policy and practice change. Evidence in these settings including but not limited to disease presentation patterns, socioeconomic characteristics of patients, responsiveness to medications, and overall outcomes would be ideal. Surveillance and registries that are set up prior to conflict settings, or early in response, would be ideal in tackling these and other key research questions [132].

Furthermore, the gap in publications on palliative care was alarming given its particular relevance and importance in settings with limited access to care for later stages of disease. This highlights the importance of developing interventions with palliative care implications as well as policies that ensure access to palliative care management such as medications to treat pain, mental health symptoms and gastrointestinal symptoms [133, 134].

### Further prioritization by policy-makers and other stakeholders on NCDs in diverse disaster settings needed

Increased understanding of the effects of diverse crises, rather than just armed conflict is also key. In Asia, the Western Pacific, and the Americas there was a specific focus on natural disasters [67, 75, 76], whereas the Eastern Mediterranean Region (EMRO) and Africa regions focused primarily on armed conflict [40, 57, 86, 97]. The effects of climate change [9], and subsequent increasing natural disasters, highlight our need to identify NCD burden in order to guide appropriate responses during these events. These feats can be achieved by greater prioritization by stakeholders that are already based in the underrepresented regions, such as development agencies and non-profits, increased political will, funding mechanism opportunities such as from the World Bank and the UNHCR, as well as through consideration by new partners establishing work in these settings.

### Further research in diverse disaster phases needed

Of note, most studies either reported on the consequences of conflict after the fact [40] or when the population of interest had relocated to a refugee camp or host country [51, 59, 62, 109]. Meanwhile, there were few studies on NCDs [44, 86, 97] affecting populations during acute crises, active conflict, or for internally displaced persons (IDPs) [51, 58, 59, 61, 62] (see Tables 6, 7, 8, 9 and 10). These findings indicate the importance of research during active crisis, including by organizations doing relief efforts such as through tracking reporting on NCD diagnoses and outcomes. While this can be challenging in such settings, it is necessary. Kohrt et al. suggest increased focuses on conducting research ethically among these vulnerable populations, as well as increased community engagement and facilitation of improved research capacity with LMIC-based partners as potential initial solutions [3]. They also encourage flexible research methods with sensitivity to these unique needs by researchers when being reviewed by funding bodies and ethics review boards [3]. Ultimately, clearly outlined policies that guide agencies responding during crises on establishing research protocols even as they provide clinical care, which are developed a priori, would be beneficial to increase the quality, rigor and ethical nature with which research can be conducted among these populations.

### Concomitant affliction with NCDs and NCD risk factors

We found that the populations studied were commonly afflicted with multiple NCD risk factors [32, 50, 53] and multiple NCDs [45, 50, 53, 83], which supports the need for consolidated care for NCDs as co-affliction confers higher risk of complications [83]. Many commonly cited risk factors in HICs such as age, family history, higher BMI, comorbid hypertension, smoking, hyperlipidemia, family history, sedentary lifestyle for DM, cancer, cardiovascular disease were cited [32, 50, 53, 64, 81, 83, 113]. However, a lack of association between NCDs and family history as well as other traditional risk factors was also found, and this may result in under-recognition and subsequent under-diagnosis in these settings [89, 135]. Packages, such as the WHO PEN, which provide a comprehensive approach to NCDs and NCD risk factors may be worthwhile when considering establishing care in these settings to ensure that NCDs are considered routinely [136]. Furthermore, an adaptation to PEN for humanitarian settings called the “PEN-H” should be considered for dissemination during crisis relief efforts [137].

### Disaster related exposures as unique contributors to NCD development and morbidity

Multiple studies identified disaster-related psychologic and physical stressors as significant risk factors for NCDs [37, 38, 41, 43, 63, 65, 78, 82, 85, 91, 92, 94], as well as described subsequent increased NCD related morbidity as a result of disaster stressors [34, 36, 43, 56, 57, 78, 95, 97]. Bereavement, injuries in the family [34, 36, 82], displacement [120], temporal/ geographical proximity [32, 56, 57, 95], and war-related physical and psychological trauma [34, 78, 94] were some of the independent predictors of diagnosis, and increased NCD morbidity [34, 36, 37, 57, 78, 82]. Refugee status was independently identified both as a risk factor for diagnosis with an NCD [32, 38, 43, 50, 69], and conferring worse morbidity as indicated by Disability Adjusted Life Years (DALYs) lost [74].

Malnutrition and food insecurity during disaster were commonly cited risk factors for increased NCD morbidity. Notably, fetal exposure to severe famine was associated with an increased risk of cancer [60], DM/impaired glucose tolerance [41, 85, 90, 92], metabolic syndrome later in life [102], and the unique phenomenon of Malnutrition Related Diabetes Mellitus (MRDM) [90]. The risk of MRDM was exacerbated by a nutritionally rich environment later in life [85, 92]. Another hypothesis for the higher prevalence of DM was lack of ability to monitor and control dietary intake and blood sugar during a crisis [38, 50, 53, 95].

Finally, environmental exposures from natural disasters [39, 42, 70, 71, 75] and war related toxins [73, 100, 105] contribute to NCD burden for these populations particularly for respiratory and cardiovascular diseases. Natural disasters impacting chronic respiratory illness include thunderstorms, earthquakes, forest fires, volcanic eruptions, and tsunami. Dust storms were a notable exception in one study [39], for which a link to increased pulmonary illness was not shown, while in contrast there was evidence of an effect on cardiovascular disease.

In sum, disaster settings confer higher incidence of NCDs and associated comorbidity. Furthermore, attention to refugee status in disaster settings is key given a disparate disease burden. Refugee populations have greater burden of disease and worsened outcomes when compared to host populations. Distribution of disease within a refugee population may be unique, and further divergent by ethnic group even among refugee populations. This is critical information to guide future humanitarian intervention design and implementation that should be sensitive to the need for tailored interventions for these sub-populations affected in that context. Furthermore, increased research is needed on the magnitude of the effect of disasters on NCD development, the development of complications of NCDs, as well as the timeliness of development of NCDs associated with exposure to disaster settings and the duration of effect.

### Overcoming barriers to management of NCD care in humanitarian crisis settings through increased health system preparedness and responsiveness

The most commonly cited barriers to healthcare access in all phases of disasters and major disease diagnoses studied, included personal attributes: low levels of education [90, 93, 138], financial difficulties [53, 93, 120], displacement [86, 93], and illiteracy [32, 50, 93]. The most commonly cited systems level concerns were lack of access to medications, and affordability of medications [53, 83, 86, 90, 93, 139]. Multiple DM specific studies noted that syringes and self-monitoring blood glucose devices were not readily available and posed a financial burden to those who required access to them [84, 86, 90]. Several studies also noted shifting of medications from the clinically indicated medication to cheaper or more available options, which may lead to worse outcomes [83, 84]. Such challenges may be magnified more for migratory refugees as compared to those who are more established in refugee camps, as demonstrated by Yusef et al. [83].

Furthermore, in many countries affected by humanitarian emergencies, there is scarce data on NCD surveillance, epidemiology, and outcomes in populations at risk in the pre-disaster setting, which creates challenges for disaster mitigation efforts [89, 99]. As a result, poorly functioning systems for delivery of NCD care [33, 86], and underdiagnosis of NCDs [89, 135] in the pre-disaster setting are compounded by new challenges resulting from widespread destruction of the health system [33, 140].

Greater attention to screening and allocation of resources to treat NCDs including acute cardiovascular events such as acute myocardial infarction and stroke are needed in disaster-prone settings, outside of other medical relief efforts. A health system situational analysis in Tunisia demonstrates the effectiveness of a robustly developing primary health care system, which falls short in the humanitarian crisis setting without established human resources, reimbursement for public sector, consensus around guidelines for management, and the absence of ancillary providers such as nutritionists or specialists for referral, when needed [35]. In post-war Liberia, with majority of CVD deaths occurring within 24 h of admission, optimization of emergency care which is the first point of contact, was also highlighted [40]. Hung et al., the only researchers focusing on the pre-hospital setting, also enforce the importance of raising awareness among first responders of the associated increased burden of NCDs during crisis and propose guidelines adapted to this [42].

Multiple studies also enforced the importance of decentralized care of NCDs from tertiary health facilities pre-disaster as this was commonly noted to hinder NCD care access during the relief phase [33, 35, 84, 86]. Several diseases including leading cancer diagnoses are amenable to prevention, screening, and early detection such as breast cancer [59, 64], cervical cancer [58, 63], and other cancers associated with tobacco use [59, 64]. Cervical cancer, for example, is amenable both to primary prevention strategies (HPV immunization and barrier protection during sexual intercourse) as well as secondary prevention (pap smears), and was identified as an opportunity for targeting by several studies with high prevalence including in Vietnam [58] and Croatia [63]. Decentralization of primary care provision to community-based settings, such as for eye care, was advocated to address the loss of healthcare infrastructure [77], and may reduce stress on facilities providing emergent care [48, 77]. Reinforcement of the public health sector’s capacity for NCD management benefits both the relief phase of disaster response as well as post-disaster rehabilitation and reconstruction [140].

Overall, increased preparedness [33, 67, 84, 86] and responsiveness by aid providers, health providers, and local governments to NCDs in disasters [67, 86] would help improve disaster mitigation assessments. Validated tools such as the WHO Stepwise approach to Surveillance [141] or Demographic Health Surveys [142] could be used for surveillance or to develop registries in countries to allow for increased pre-disaster preparedness. Finally, healthcare systems can address the imminent need for palliative interventions that aim to reduce excess morbidity and suffering from NCDs [133].

### Methodology and research infrastructure also key

As further guidance, we wanted to comment on study design as a key focus for future studies in this setting. The predominant study design consisted of retrospective chart reviews [36, 40, 42, 46, 49] with a minority of cross-sectional studies [39, 47, 51, 59, 64]. We observed that several studies either did not include a comparison group in their study design, or they used a time period across which the comparison was made that was arbitrary in nature. While challenging to conduct given the context of the studies, this limits validity of findings in many studies [52, 63–65]. These predominant research designs limit the ability to draw conclusions for causation, or to accurately measure the effects of disaster itself, although associations have been noted as previously stated under the discussion heading on disaster related exposures. In future, cohort study designs, as well as potential registries [132] alongside other prospective studies would enrich current knowledge on NCD determinants in disaster settings. Additionally, these study designs would enable better assessment of the long-term effects and complications of NCDs in these settings that are potentially exacerbated by the disaster context.

In addition, it was noted that publications were clustered by research group or author [45, 55, 85, 102, 104, 109–111, 143], which speaks to the need for increased academic outputs in LMICs, and Africa in particular. Increased infrastructure and capacity on research development is needed, including support for agencies at the front-lines implementing and delivering clinical programs, and who have the potential to concomitantly implement effective research on target populations or their programs.

Finally, several articles that were included in our results included NCDs as a peripheral focus, rather than as primary outcomes [77, 135]. Consideration should be given to include comparison or control groups in study design, for example individuals in neighboring regions, non-refugee counterparts, or matched sample populations not afflicted by the disease [34, 58] to be able to better assess and thus delineate the effects of the humanitarian crises itself on disease outcomes. Additionally, long-term cohorts and registries [109, 111] would be ideal to better understand the diversity of diseases and contributory factors in even greater depth. Of all the studies included, none referred to the Sphere guidelines [144], WHO Noncommunicable Diseases in Emergencies brief [145], or WHO PEN package of essential NCD interventions [136] as markers for study design, which we propose be included in future research.

### Limitations

The last date of publication submission included in our findings is 2017, which limits conclusions based on findings published thereafter. With that said, we have demonstrated inciting evidence to guide future research, including through summarizing trends over the past two decades (see Fig. 2), and highlighting key findings from existing literature during that window that includes a predominant focus on cancer, and less focus on cardiovascular disease which carries a predominant NCD burden globally, a relative lack of focus on chronic respiratory disease and pediatric populations among other key findings. In turn, we have followed the protocol outlined and pre-published in PROSPERO demonstrating the novelty of this comprehensive review on NCDs in humanitarian settings that has not been undertaken as yet. In addition, although we were inclusive of major languages spoken in our search strategy (specifically English, French and Arabic) if the articles were not indexed using English words, or in the databases utilized, they would not have been included in our results. We are cognizant of the limitations of publication bias, and support continued advocacy for representation of various languages in primary research, journals and popular databases. All the same, we trust that despite these limitations the findings will contribute to increasing prioritization of NCDs in humanitarian settings, stimulate research ideas, and engage policy-makers at the country, national and international level.

## Conclusion

An increased focus on the effects of, and mitigating factors for, NCDs occurring in disaster-afflicted LMICs is direly needed. While majority of studies included in our review presented epidemiologic evidence for the burden of disease, research is needed to address contributing factors, and means of managing disease in these extremely resource-variable settings. Regions particularly lacking evidence on LMICs in our study were Africa and the Americas; majority of evidence was from the EMRO region. Among the four lead NCDs, chronic respiratory disease was under-addressed despite evidence that it contributes to high morbidity in crisis. Furthermore, increased evidence on actual diseases such as myocardial infarction and diabetes, rather than simply focusing on risk factors such as hypertension is also needed with greater understanding of NCD epidemiology to guide allocation of resources and policy-makers. Attention to vulnerable populations including women and refugees is also a priority. Refugees have unique exposures that may predispose them to certain illnesses, such as MRDM, and management needs that warrant separate attention from host populations. Given this, we propose that refugee status be considered as an independent risk factor for future studies and interventions. All in all, screening and prevention for NCDs should be a priority alongside communicable disease programs, such as counseling for smoking cessation, counseling on diet, HPV vaccination, and screening for common cancers like breast and cervical cancer. Studies on implementation for these and other interventions will be key, and the use of implementation science to guide design and assess feasibility could be useful in these challenging settings. Additionally, policies allocating resources to equip health systems to address NCDs both pre-disaster and during crisis will enhance these efforts, such as through decentralization of care from tertiary settings that are already overextended during crisis. Finally, the need to address disease in disaster settings in collaboration with LMIC-based partners, community members, as well as other sectors outside of health silos such as agriculture and urban policy-makers, was also supported.

## Supplementary Information

**Additional file 1: **Quality Assessment (Method: Newcastle-Ottawa Quality Assessment Scale for case control studies/ cohort studies – latter in bold).

## Data Availability

All data generated or analyzed during this study are included in this published article and its supplementary information files. The study is registered at PROSPERO (CRD42018088769).

## Abbreviations

AMI: Acute myocardial infarction
BMI: Body mass index
CAT: COPD Assessment Tool
COPD: Chronic obstructive pulmonary disease
CVD: Cardiovascular disease
DALYs: Disability adjusted life years
DM: Diabetes mellitus
ECC: Exceptional care committee
EMRO: Eastern Mediterranean Region
HICs: High income countries
HPV: Human papillomavirus
HRQOL: Health-related quality of life
HTN: Hypertension
ICRC: International Committee of the Red Cross
IFG: Impaired fasting glucose
LDL: Low density lipoprotein
LMICs: Low and middle-income countries
NCDs: Non-communicable diseases
MRDM: Malnutrition related diabetes mellitus
MeSH: Medical Subject Heading
NGOs: Non-governmental organisations
OR: Odds ratio
SEA: South East Asia
UNHCR: United Nations High Commissioner for Refugees
UNISDR: United Nations Office for Disaster Risk Reduction
UNRWA: United Nations Relief and Works Agency
WHO: World health organization
WHOQOL-BREF: World Health Organization Quality of Life Questionnaire
WP: Western Pacific
