# Supplementary material for: Systematic review on chronic non-communicable disease in disaster settings
Source: BMC Public Health. 2022 Jun 21;22:1234. doi: 10.1186/s12889-022-13399-z (PMC9210736; doi:10.1186/s12889-022-13399-z)
Supplement: Supplementary file 1 — Additional file 1: Quality Assessment (Method: Newcastle-Ottawa Quality Assessment Scale for case control studies/ cohort studies – latter in bold). [file 12889_2022_13399_MOESM1_ESM.docx]

**Additional file 1**

**Quality Assessment (Method: Newcastle-Ottawa Quality Assessment Scale for case control studies/ cohort studies – latter in bold)**

|  | Case Definition Adequate | **Representativeness of cases/ exposed cohort** | **Selection of control/ non-exposed cohort** | Definition of controls | **Comparability: based on design or analysis** | **Ascertainment of exposure** | Cases and controls: same ascertainment method | Cases and controls: same non-response rate | **Outcome of interest not present at start of study** | **Assessment of Outcome** | **Follow-up long enough for outcomes** | **Adequacy of follow up** |
| --- | --- | --- | --- | --- | --- | --- | --- | --- | --- | --- | --- | --- |
| Cardiovascular disease | | | | | | | | | | | | |
| **Abukhdeir (2013) (37)** |  | Truly representative (*) | Same community (*) |  | Yes (**) | Structured interview (*) |  |  | Yes (*) | Self report | NA (cross-sectional) | NA (cross-sectional) |
| **Ahmad (2015) (106)** |  | Selected group | NA (cross-sectional, descriptive) |  | NA (cross-sectional, descriptive) | Interview not blinded |  |  | No | Self report | NA (cross-sectional) | NA (cross-sectional) |
| **Armenian (1998) (53)** | Self report | Truly representative (*) | Community controls (*) | No history of disease (*) | Yes (**) | No description | Yes (*) | Same rate (*) |  |  |  |  |
| **Ben Romdhane (2015) (107)** |  | Selected group | NA (cross-sectional, descriptive) |  | NA (cross-sectional, descriptive) | Interview not blinded |  |  | Yes (*) | Self report | NA (cross-sectional) | NA (cross-sectional) |
| **Bergovec (2005) (57)** |  | Somewhat representative (*) | Same community (*) |  | Yes (**) | Structured interview (*) |  |  | Yes (*) | Self report | Yes (*) | No statement |
| **Chen (2009) (54)** |  | Selected group | No description (case series) |  | NA (case series) | Structured interview (*) |  |  | Yes (*) | Self report | Yes (*) | Complete follow up (*) |
| **Ebling (2007) (63)** |  | Somewhat representative (*) | Drawn from a different source |  | Yes (**) | Both secure record and written self report (*) |  |  | Yes (*) | Record linkage (*) | Yes (*) | Complete follow up (*) |
| **Ebrahimi (2014) (56)** |  | Truly representative (*) | Same community (*) |  | NA (multiple cross-sectional data points, single group cohort) | Secure record (*) |  |  | Yes (*) | Record linkage (*) | Yes (*) | No statement |
| **Huerga (2009) (36)** |  | Somewhat representative (*) | Same community (*) |  | NA (retrospective, single group cohort) | Secure record (*) |  |  | Yes (*) | Record linkage (*) | Yes (*) | No statement |
| **Hult (2010) (59)** |  | Somewhat representative (*) | Same community (*) |  | Yes (**) | Structured interview (*) |  |  | Yes (*) | Record linkage (*) | Yes (*) | Complete follow up (*) |
| **Hung (2013) (41)** |  | Selected group | Same community (*) |  | NA (retrospective, single group cohort) | Secure record (*) |  |  | No | Record linkage (*) | Yes (*) | Subjects lost to follow up unlikely to introduce bias – small number lost (*) |
| **Kadojic (1999) (40)** |  | Selected group | Same community (*) |  | Yes (**) | Structured interview (*) |  |  | No | Record linkage (*) | NA (cross-sectional) | No statement |
| **Kallab (2015) (32)** |  | Selected group | Same community (*) |  | NA (multiple cross-sectional data points, single group cohort) | Structured interview (*) |  |  | No | Self report | Yes (*) | No statement |
| **Khader (2014) (47)** |  | Truly representative (*) | Same community (*) |  | NA (retrospective, single group cohort) | Secure record (*) |  |  | Yes (*) | Record linkage (*) | Yes (*) | Follow up rate >20% |
| **Marjanovic (2003) (39)** |  | Somewhat representative (*) | Same community (*) |  | NA (cross-sectional, descriptive) | Secure record (*) |  |  | Yes (*) | Record linkage (*) | NA (cross-sectional) | NA (cross-sectional) |
| **Markoglou (2005) (43)** |  | Truly representative (*) | NA (cross-sectional, descriptive) |  | NA (cross-sectional, descriptive) | Secure record (*) |  |  | Yes (*) | Record linkage (*) | NA (cross-sectional) | NA (cross-sectional) |
| **Mateen (2012) (46)** |  | Somewhat representative (*) | NA (cross-sectional, descriptive) |  | NA (cross-sectional, descriptive) | Secure record (*) |  |  | Yes (*) | Record linkage (*) | NA (cross-sectional) | NA (cross-sectional) |
| **Miric (2001) (52)** |  | Truly representative (*) | NA (retrospective chart review) |  | NA (retrospective chart review) | Secure record (*) |  |  | Yes (*) | Record linkage (*) | NA (retrospective chart review) | NA (retrospective chart review) |
| **Mousa (2010) (44)** |  | Somewhat representative (*) | No description (case series) |  | NA (case series) | Structured interview (*) |  |  | Yes (*) | Self report | NA (no follow up) | NA (no follow up) |
| **Otoukesh (2012) (48)** |  | Somewhat representative (*) | NA (cross-sectional, descriptive) |  | NA (cross-sectional, descriptive) | Secure record (*) |  |  | Yes (*) | Record linkage (*) | Yes (*) | NA (cross-sectional) |
| **Sibai (2001) (33)** |  | Somewhat representative (*) | NA (cross-sectional, descriptive) |  | NA (cross-sectional, descriptive) | Self-report |  |  | Yes (*) | Self-report | Yes (*) | Small % lost to follow up, unlikely to introduce bias (*) |
| **Sibai (2007) (45)** |  | Truly representative (*) | NA (no control group) |  | NA (retrospective, single group cohort) | Structured interview (*) |  |  | Yes (*) | Self report | Yes (*) | Complete follow up (*) |
| **Strong (2015) (34)** |  | Selected group | NA (cross-sectional, descriptive) |  | NA (cross-sectional, descriptive) | Structured interview (*) |  |  | No | Self report | NA (cross-sectional) | NA (cross-sectional |
| **Sun (2013) (42)** |  | Truly representative (*) | NA (cross-sectional, descriptive) |  | NA (cross-sectional, descriptive) | Structured interview (*) |  |  | Yes (*) | Independent assessment | NA (cross-sectional) | NA (cross-sectional) |
| **Tomic (2009) (44)** | Yes, with validation (*) | Obviously representative (*) | Hospital controls (*) | No history of disease (*) | Yes (**) | No description | Yes (*) | Same rate (*) |  |  |  |  |
| **Vasilj (2006) (51)** |  | Truly representative (*) | NA (retrospective chart review) |  | NA (retrospective chart review) | Secure record (*) |  |  | Yes (*) | Record linkage (*) | NA (retrospective chart review) | NA (retrospective chart review) |
| **Vukovic (2005) (48)** |  | Truly representative (*) | NA (retrospective chart review) |  | NA (retrospective chart review) | Secure record (*) |  |  | Yes (*) | Record linkage (*) | NA (retrospective chart review) | NA (retrospective chart review) |
| **Yusef (2000) (35)** |  | Truly representative (*) | NA (cross-sectional, descriptive) |  | NA (cross-sectional, descriptive) | Secure record (*) |  |  | No | Record linkage (*) | NA (retrospective chart review) | NA (retrospective chart review) |
| **Zubaid (2006) (58)** | Yes, record linkage | Truly representative (*) | Hospital controls (*) | No history of disease (*) | NA (retrospective chart review) | No description | Yes (*) | Same rate (*) |  |  |  |  |
| Cancer | | | | | | | | | | | | |
| **Huynh (2004) (70)** | Yes, record linkage | Truly representative (*) | Hospital controls (*) | No history of disease (*) | NA (retrospective chart review) | No description | Yes (*) | Non-respondents described |  |  |  |  |
| **Khan (1997) (72)** |  | Truly representative (*) | NA (cross-sectional, descriptive) |  | NA (cross-sectional, descriptive) | Secure record (*) |  |  | Yes (*) | Record linkage (*) | NA (cross-sectional) | NA (cross-sectional) |
| **Li (2012) (67)** |  | Truly representative (*) | NA (retrospective chart review) |  | NA (retrospective chart review) | Secure record (*) |  |  | Yes (*) | Record linkage (*) | NA (retrospective chart review) | NA (retrospective chart review) |
| **Marom (2014) (61)** |  | Truly representative (*) | No description (case series) |  | NA (case series) | Secure record (*) |  |  | Yes (*) | Record linkage (*) | NA (retrospective chart review) | NA (retrospective chart review) |
| **McKenzie (2015) (64)** |  | Truly representative (*) | NA (retrospective chart review) |  | NA (retrospective chart review) | Secure record (*) |  |  | Yes (*) | Record linkage (*) | NA (retrospective chart review) | NA (retrospective chart review) |
| **Milojkovic (2005) (68)** |  | Truly representative (*) | NA (retrospective chart review) |  | NA (retrospective chart review) | Secure record (*) |  |  | Yes (*) | Record linkage (*) | NA (retrospective chart review) | NA (retrospective chart review) |
| **Otoukesh (2012) (48)** |  | Somewhat representative (*) | NA (cross-sectional, descriptive) |  | NA (cross-sectional, descriptive) | Secure record (*) |  |  | Yes (*) | Record linkage (*) | Yes (*) | NA (cross-sectional) |
| **Shamseddine (2004) (66)** |  | Truly representative (*) | NA (cross-sectional, descriptive) |  | NA (cross-sectional, descriptive) | Secure record (*) |  |  | Yes (*) | Record linkage (*) | NA (cross-sectional) | NA (cross-sectional) |
| **Sibai (2001) (33)** |  | Somewhat representative (*) | NA (cross-sectional, descriptive) |  | NA (cross-sectional, descriptive) | Self-report |  |  | Yes (*) | Self-report | Yes (*) | Small % lost to follow up, unlikely to introduce bias (*) |
| **Telarovic (2006) (69)** |  | Selected group | NA (cross-sectional, descriptive) |  | NA (cross-sectional, descriptive) | Secure record (*) |  |  | Yes (*) | Record linkage (*) | Yes (*) | NA (cross-sectional) |
| Chronic respiratory disease | | | | | | | | | | | | |
| **Abul (2001) (73)** |  | Selected group | NA (cross-sectional, descriptive) |  | NA (cross-sectional, descriptive) | Secure record (*) |  |  | No | Record linkage (*) | NA (cross-sectional) | NA (cross-sectional) |
| **Bijani (2002) (75)** |  | Selected group | NA (cross-sectional, descriptive) |  | NA (cross-sectional, descriptive) | Structured interview (*) |  |  | Yes (*) | Structured interview (*) | NA (cross-sectional) | NA (cross-sectional) |
| **Ebrahimi (2014) (56)** |  | Truly representative (*) | Same community (*) |  | NA (multiple cross-sectional data points, single group cohort) | Secure record (*) |  |  | Yes (*) | Record linkage (*) | Yes (*) | No statement |
| **El-Sharif (2002) (83)** |  | Truly representative (*) | NA (cross-sectional, descriptive) |  | NA (cross-sectional, descriptive) | Written self-report |  |  | Yes (*) | Self report | NA (cross-sectional) | NA (cross-sectional) |
| **Forouzan (2014) (78)** |  | Selected group | NA (cross-sectional, descriptive) |  | NA (cross-sectional, descriptive) | Structured interview (*) |  |  | No | Structured interview (*) | NA (cross-sectional) | Significant loss to follow up |
| **Hung (2013) (41)** |  | Selected group | Same community (*) |  | NA (retrospective, single group cohort) | Secure record (*) |  |  | No | Record linkage (*) | Yes (*) | Subjects lost to follow up unlikely to introduce bias – small number lost (*) |
| **Kunii (2002) (79)** |  | Somewhat representative (*) | NA (cross-sectional, descriptive) |  | NA (cross-sectional, descriptive) | Structured interview (*) |  |  | Yes (*) | Record linkage (*) | NA (cross-sectional) | NA (cross-sectional) |
| **Lari (2014) (77)** |  | Selected group | NA (cross-sectional, descriptive) |  | NA (cross-sectional, descriptive) | Structured interview |  |  | Yes (*) | Structured interview (*)_ | NA (cross-sectional) | NA (cross-sectional |
| **Mirsadraee (2011) (76)** | Yes, with independent validation (*) | Potential for selection bias not stated. | No description | No history of exposure (*) | No, based on analysis. | Secure record (*) | Yes (*) | No description |  |  |  |  |
| **Molla (2014) (84)** |  | Truly representative (*) | Drawn from a different source |  | No, based on analysis. | Secure record (*) |  |  | Yes (*) | Structured interview (*) | NA (cross sectional) | NA (cross sectional) |
| **Naumova (2007) (80)** |  | Somewhat representative (*) | NA (cross-sectional, descriptive) |  | NA (cross-sectional, descriptive) | Secure record (*) |  |  | Yes (*) | Record linkage (*) | NA (cross sectional) | NA (cross sectional) |
| **Guha Sapir (2007) (81)** |  | Selected group | NA (cross-sectional, descriptive) |  | NA (cross sectional, descriptive) | Secure record (*) |  |  | Yes (*) | Record linkage (*) | NA (cross-sectional) | NA (cross-sectional) |
| **RedwoodCampbell (2006) (82)** |  | Selected group | NA (cross-sectional, descriptive |  | NA (cross sectional, descriptive) | Secure record (*) |  |  | Yes (*) | Record linkage (*) | NA (cross-sectional) | NA (cross-sectional |
| **Wright (2010) (74)** |  | Truly representative (*) | NA (cross-sectional, descriptive) |  | NA (cross sectional, descriptive) | Secure record (*) |  |  | Yes (*) | Structured interview (*) | NA (cross sectional) | NA (cross sectional) |
| Diabetes | | | | | | | | | | | | |
| **Abukhdeir (2013) (37)** |  | Truly representative (*) | Same community (*) |  | Yes (**) | Structured interview (*) |  |  | Yes (*) | Self report | NA (cross sectional) | NA (cross sectional) |
| **Ahmad (2015) (106)** |  | Selected group | NA (cross-sectional, descriptive) |  | NA (cross-sectional, descriptive) | Interview not blinded |  |  | No | Self report | NA (cross-sectional) | NA (cross-sectional) |
| **Alabed (2014) (100)** |  | Somewhat representative (*) | NA (cross-sectional, descriptive) |  | NA (cross sectional, descriptive) | Structured interview (*) |  |  | Yes (*) | Structured interview (*) | NA (cross sectional) | NA (cross sectional) |
| **Ali-Shtayeh (2012) (102)** |  | Somewhat representative (*) | NA (cross-sectional, descriptive) |  | NA (cross sectional descriptive) | Secure record (*) |  |  | Yes (*) | Self-report (*) | NA (cross sectional) | NA (cross sectional) |
| **AlKasseh (2013) (105)** | Self report | Obviously representative (*) | Community controls (*) | No history of disease (*) | Yes (**) | No description | Yes (*) | Same rate (*) |  |  |  |  |
| **An (2014) (85)** |  | Truly representative (*) | Same community (*) |  | Yes (**) | Written self report |  |  | Yes (*) | Record linkage (*) | Yes (*) | Complete follow up (*) |
| **Armenian (1998) (53)** | Self report | Truly representative (*) | Community controls (*) | No history of disease (*) | Yes (**) | No description | Yes (*) | Same rate (*) |  |  |  |  |
| **Balabanova (2009) (94)** |  | Selected group | NA (cross-sectional, descriptive) |  | NA (cross-sectional, descriptive) | Interview not blinded |  |  | Yes (*) | Self report | NA (cross-sectional) | NA (cross-sectional) |
| **Ben Romdhane (2015) (107)** |  | Selected group | NA (cross-sectional, descriptive) |  | NA (cross-sectional, descriptive) | Interview not blinded |  |  | Yes (*) | Self report | NA (cross-sectional) | NA (cross-sectional) |
| **Besancon (2015) (60)** |  | No description of the derivation of the cohort | NA (cross-sectional, descriptive) |  | NA (cross-sectional, descriptive) | No description |  |  | Yes (*) | Self report | NA (cross-sectional) | NA (cross-sectional) |
| **Ebling (2007) (63)** |  | Somewhat representative (*) | Drawn from a different source |  | Yes (**) | Both secure record and written self report (*) |  |  | Yes (*) | Record linkage (*) | Yes (*) | Complete follow up (*) |
| **Eljedi (2006) (88)** |  | Truly representative (*) | Same community (*) |  | Yes (**) | Written self report |  |  | Yes (*) | Self report | Yes (*) | Complete follow up (*) |
| **Gilder (2014) (99)** |  | Somewhat representative (*) | NA (cross-sectional, descriptive) |  | NA (cross-sectional, descriptive) | Secure record (*) |  |  | Yes (*) | Record linkage (*) | NA (cross-sectional) | NA (cross-sectional) |
| **Habtu (1999) (92)** |  | Somewhat representative (*) | NA (cross-sectional, descriptive) |  | NA (cross-sectional, descriptive) | Secure record (*) |  |  | Yes (*) | Record linkage (*) | NA (cross-sectional) | NA (cross-sectional) |
| **Hult (2010) (59)** |  | Somewhat representative (*) | Same community (*) |  | Yes (**) | Structured interview (*) |  |  | Yes (*) | Record linkage (*) | Yes (*) | Complete follow up (*) |
| **Kallab (2015) (32)** |  | Selected group | Same community (*) |  | NA (multiple cross-sectional data points, single group cohort) | Secure record and structured interview (*) |  |  | No | Record linkage and self report (*) | Yes (*) | No statement |
| **Karrouri (2014) (86)** |  | Selected group | NA (cross-sectional, descriptive) |  | NA (cross-sectional, descriptive) | Secure record (*) |  |  | Yes (*) | Record linkage (*) | NA (cross-sectional) | NA (cross-sectional) |
| **Khader (2012) (98)** |  | Somewhat representative (*) | Same community (*) |  | Yes (**) | Secure record (*) |  |  | Yes (*) | Record linkage (*) | Yes (*) | Subjects lost to follow up unlikely to introduce bias - small number lost (*) |
| **Khader  (2013) (95)** |  | Somewhat representative (*) | Same community (*) |  | Yes (**) | Secure record (*) |  |  | Yes (*) | Record linkage (*) | Yes (*) | Subjects lost to follow up unlikely to introduce bias - small number lost (*) |
| **Khader  (2014) (96)** |  | Somewhat representative (*) | Same community (*) |  | Yes (**) | Secure record (*) |  |  | Yes (*) | Record linkage (*) | Yes (*) | Subjects lost to follow up unlikely to introduce bias - small number lost (*) |
| **Khader (2014) (97)** |  | Somewhat representative (*) | Same community (*) |  | Yes (**) | Secure record (*) |  |  | Yes (*) | Record linkage (*) | Yes (*) | Subjects lost to follow up unlikely to introduce bias - small number lost (*) |
| **Li (2010) (90)** |  | Somewhat representative (*) | Same community (*) |  | Yes (**) | Secure record (*) |  |  | Yes (*) | Record linkage (*) | Yes (*) | Complete follow up (*) |
| **Lumey (2015) (91)** |  | Somewhat representative (*) | Drawn from a different source |  | Yes (**) | Both secure record and written self report (*) |  |  | Yes (*) | Record linkage (*) | Yes (*) | Complete follow up (*) |
| **Mansour (2008) (93)** |  | Selected group | NA (cross-sectional, descriptive) |  | NA (cross-sectional, descriptive) | Both secure record and written self report (*) |  |  | Yes (*) | Record linkage (*) | NA (cross-sectional) | NA (cross-sectional) |
| **Mateen (2012) (46)** |  | Somewhat representative (*) | NA (cross-sectional, descriptive) |  | NA (cross-sectional, descriptive) | Secure record (*) |  |  | Yes (*) | Record linkage (*) | NA (cross-sectional) | NA (cross-sectional) |
| **Mousa (2010) (44)** |  | Somewhat representative (*) | No description (case series) |  | NA (case series) | Structured interview (*) |  |  | Yes (*) | Self report | NA (no follow up) | NA (no follow up) |
| **Ramachandran (2006) (87)** | Yes (*) | Obviously representative (*) | Community controls (*) | No history of disease (*) | Yes (*) | Interview not blinded | Yes (*) | Same rate (*) |  |  |  |  |
| **Read (2015) (62)** |  | Somewhat representative (*) | NA (cross-sectional, descriptive) |  | NA (cross-sectional, descriptive) | Secure record (*) |  |  | Yes (*) | Record linkage (*) | NA (cross-sectional) | NA (cross-sectional) |
| **Sengul (2004) (89)** |  | Somewhat representative (*) | Same community (*) |  | Yes (*) | Secure record (*) |  |  | Yes (*) | Record linkage (*) | Yes (*) | Complete follow up (*) |
| **Sofeh (2004) (103)** |  | Somewhat representative (*) | NA (cross-sectional, descriptive) |  | NA (cross-sectional, descriptive) | Secure record (*) |  |  | Yes (*) | Record linkage (*) | NA (cross-sectional) | NA (cross-sectional) |
| **Strong (2015) (34)** |  | Selected group | NA (cross-sectional, descriptive) |  | NA (cross-sectional, descriptive) | Structured interview (*) |  |  | No | Self report | NA (cross-sectional) | NA (cross-sectional |
| **Wagner (2016) (65)** |  | Selected group | Same community (*) |  | Yes (*) | Structured interview (*) |  |  | Yes (*) | Pre test / post-test | Yes (*) | Complete follow up (*) |
| **Yaghi (2012) (101)** |  | Somewhat representative (*) | NA (cross-sectional, descriptive) |  | NA (cross-sectional, descriptive) | Secure record (*) |  |  | Yes (*) | Record linkage (*) | NA (cross-sectional) | NA (cross-sectional) |
| **Yusef (2000) (35)** |  | Truly representative (*) | NA (cross-sectional, descriptive) |  | NA (cross-sectional, descriptive) | Secure record (*) |  |  | No | Record linkage (*) | NA (retrospective chart review) | NA (retrospective chart review) |
| Other Non-Communicable Diseases | | | | | | | | | | | | |
| **Amini (2010) (111)** |  | Selected group | NA (cross-sectional, descriptive) |  | NA (cross-sectional, descriptive) | Structured interview (*) |  |  | Yes (*) | Self report | NA (cross-sectional) | NA (cross-sectional) |
| **Armenian (1998) (53)** | Self report | Truly representative (*) | Community controls (*) | No history of disease (*) | Yes (**) | No description | Yes (*) | Same rate (*) |  |  |  |  |
| **Chan (2010) (113)** |  | Somewhat representative (*) | NA (cross-sectional, descriptive) |  | NA (cross-sectional, descriptive) | Structured interview (*) |  |  | Yes (*) | Self report | NA (cross-sectional) | NA (cross-sectional) |
| **Chan (2009) (115)** |  | Somewhat representative (*) | NA (cross-sectional, descriptive) |  | NA (cross-sectional, descriptive) | Structured interview (*) |  |  | Yes (*) | Self report | NA (cross-sectional) | NA (cross-sectional) |
| **Hung (2013) (41)** |  | Somewhat representative (*) | NA (cross-sectional, descriptive) |  | NA (cross-sectional, descriptive) | Secure record (*) |  |  | Yes (*) | Record linkage (*) | NA (cross-sectional) | NA (cross-sectional) |
| **Khateri (2003) (110)** |  | Somewhat representative (*) | NA (cross-sectional, descriptive) |  | NA (cross-sectional, descriptive) | Secure record |  |  | Yes (*) | Record linkage (*) | Yes (*) | NA (cross-sectional) |
| **Leeuw (2014) (112)** |  | Somewhat representative (*) | NA (cross-sectional, descriptive) |  | NA (cross-sectional, descriptive) | Self-report |  |  | Yes (*) | Self-report | Yes (*) | NA (cross-sectional) |
| **Li (2011) (137)** |  | Somewhat representative (*) | NA (cross-sectional, descriptive) |  | NA (cross-sectional, descriptive) | Secure record |  |  | Yes (*) | Record linkage (*) | Yes (*) | NA (cross-sectional) |
| **Mateen (2012) (46)** |  | Somewhat representative (*) | NA (cross-sectional, descriptive) |  | NA (cross-sectional, descriptive) | Secure record (*) |  |  | Yes (*) | Record linkage (*) | NA (cross-sectional) | NA (cross-sectional) |
| **Mateen (2012) (109)** |  | Somewhat representative (*) | NA (cross-sectional, descriptive) |  | NA (cross-sectional, descriptive) | Secure record (*) |  |  | Yes (*) | Record linkage (*) | Yes (*) | NA (cross-sectional) |
| **McKenzie (2015) (64)** |  | Somewhat representative (*) | NA (cross-sectional, descriptive) |  | NA (cross-sectional, descriptive) | Secure record (*) |  |  | Yes (*) | Record linkage (*) | Yes (*) | NA (cross-sectional) |
| **Otoukesh (2012) (48)** |  | Somewhat representative (*) | NA (cross-sectional, descriptive) |  | NA (cross-sectional, descriptive) | Secure record (*) |  |  | Yes (*) | Record linkage (*) | Yes (*) | NA (cross-sectional) |
| **RedwoodCampbell (2006) (82)** |  | Selected group | NA (cross-sectional, descriptive |  | NA (cross sectional, descriptive) | Secure record (*) |  |  | Yes (*) | Record linkage (*) | Yes (*) | NA (cross-sectional |
| **Sibai (2001) (33)** |  | Somewhat representative (*) | NA (cross-sectional, descriptive) |  | NA (cross-sectional, descriptive) | Self-report |  |  | Yes (*) | Self-report | Yes (*) | Small % lost to follow up, unlikely to introduce bias (*) |
| **Strong (2015) (34)** |  | Selected group | NA (cross-sectional, descriptive) |  | NA (cross-sectional, descriptive) | Structured interview (*) |  |  | No | Self-report | Yes (*) | NA (cross-sectional) |
